# Engineering of Optical and Electrical Properties of Electrodeposited Highly Doped Al:ZnO and In:ZnO for Cost-Effective Photovoltaic Device Technology

**DOI:** 10.3390/mi13111966

**Published:** 2022-11-13

**Authors:** Dimitra N. Papadimitriou

**Affiliations:** National Technical University of Athens, Heroon Polytechniou 9, GR-15780 Athens, Greece; dnp.ntua@gmail.com; Tel.: +30-697-414-9515

**Keywords:** Al:ZnO, In:ZnO, ECD processing, optical properties, electrical properties, Burstein–Moss effect, carrier concentration, CIS/CIGS photovoltaic

## Abstract

Resistivity and transparency of zinc-oxide layers (ZnO) for chalcopyrite photovoltaic technology applications were engineered by activation of the Burstein–Moss (BM) effect at high concentrations of aluminium (Al) and indium (In) dopant. The Al:ZnO and In:ZnO layers were processed by cost-effective, large-area, fast-rate electrochemical deposition techniques from aqueous solution of zinc nitrate (Zn(NO_3_)_2_) and dopant trichlorides, at negative electrochemical potential of E_C_ = (−0.8)–(−1.2) V, moderate temperature of 80 °C, and solute dopant concentrations of AlCl_3_ and InCl_3_ up to 20 and 15 mM, respectively. Both Al:ZnO and In:ZnO layers were deposited on Mo/glass substrates with ZnO and ZnO/ZnSe buffers (Al:ZnO/ZnO/Mo/glass, In:ZnO/ZnO/ZnSe/Mo/glass), respectively. Based on the band-gap energy broadening of Al:ZnO and In:ZnO originated by the BM effect, maximum carrier concentrations of the order 10^20^ and 10^21^ cm^−3^, respectively, were determined by optical characterization techniques. The (electrical) resistivity values of Al:ZnO calculated from optical measurements were commensurate with the results of electrical measurements (10^−4^ Ohm·cm). In both cases (Al:ZnO and In:ZnO), calibration of carrier density in dependence of solute dopant concentration (AlCl_3_ and InCl_3_) was accomplished. The p–n junctions of Au/In:ZnO/ZnO/ZnSe/CIGS/Mo on glass substrate exhibited current–voltage (I–V) characteristics competing with those of crystalline silicon (c-Si) solar cells.

## 1. Introduction

The field of ZnO research and applications has widely been scanned, in the past and at present, as already reported in our recent publications [1,2,3,4]. Our efforts are primarily focused on the engineering of the structural, optical, and electrical properties of chalcopyrite semiconductor based thin-film solar cells (TFSCs) [5,6,7,8,9,10,11,12,13] with Cu(In,Ga)Se_2_ (CIGS) absorber [1], ZnSe buffer [4], and ZnO window, front-contact, and antireflective coating (ARC) [2,3] processed by electrochemical deposition (ECD) techniques. Electrodeposition with cost-effective, large-area, moderate-temperature, fast-rate performance can be scaled-up to industrial processes. Overall processing of CIGS chalcopyrite selenide absorber [1], ZnSe buffer with alternate cubic/sphalerite and hexagonal/wurtzite structure to relax (elastic) strain/stress [4] in the layer sequence, ZnO window of highly to ultra-highly doped ZnO bilayer [3] to effectively accelerate and collect carriers, and ZnO-Nanorod (ZnO-NR) ARC [2] by ECD is targeted to overcome current process incompatibilities. Common disadvantages of flow-oriented production of commercialized CuInS_2_ (CIS) and CIGS TFSCs result mainly from the simultaneous use of moderate temperature (50–70 °C) non-vacuum and high-temperature (500–700 °C) vacuum processes applied to the absorber (multi-step evaporation at elevated temperatures [14,15]), buffer (chemical bath deposition at moderate temperatures [16,17,18,19,20]), and window (sputtering at high temperatures [21,22,23,24,25,26]) layers.

Most of the group II–VI binary compound semiconductors crystallize in either cubic/sphalerite (zinc-blende type) or hexagonal/wurtzite structure. ZnO forms mostly in the wurtzite (WZ) crystal structure with in-plane and normal-to-the-plane lattice constants of a = 3.25(495) Å and c = 5.21(069) Å, respectively [27]. ZnO has attracted attention by its prospects for optoelectronic applications owing to its direct, dipole-allowed wide band-gap of E_g_ (300 K) = 3.4 eV, in the short-wavelength region, and its relatively large exciton binding energy of 60 meV [28,29]. The group-II oxide and the group-III nitride semiconductors have direct band-gaps, which cover the ultraviolet (UV) to infrared (IR) energy range. Particularly, the wide band-gaps of AlN (6.2 eV), GaN (3.42 eV), and ZnO (3.37 eV) are favorable for high-power, short-wavelength light emitting devices [30,31,32]. Several optoelectronic applications of ZnO overlap with those of GaN, which is widely used for the production of green, blue-ultraviolet, and white light-emitting devices [33]. However, the interest in the ZnO research and applications has been facilitated due to its large exciton binding energy, paving the way for efficient room-temperature exciton based emitters, and its sharp optical transitions, enabling low-threshold laser diodes [28]. The fact that ZnO can be synthesized in nanostructured form has proven to be of great significance in the development of nanoscale p–n junctions that can sufficiently increase the injection rate of carriers [2]. Moreover, the availability of fairly good-quality single-crystal ZnO and a much simpler crystal-growth technology resulted in potentially lower costs for ZnO- compared to GaN-based devices. In comparison with GaN, the ZnO advantages are thus availability of a native substrate, relative ease of wet chemical etching for device fabrication, much higher free exciton binding energy (60 meV) than that of GaN (21–25 meV), and biexciton binding energies in the order of the 25 meV thermal energy at room-temperature. The ZnO technology interest was amplified by reports of p-type conduction and ferromagnetic behavior when doped with transition metals. ZnO has been predicted to exhibit ferromagnetic behavior at room-temperature (RT) via a hole-mediated exchange mechanism [34]. Stable p-type behavior is thus a requirement for the successful demonstration of ZnO spintronics in electronic and photonic applications.

The band-gap of ZnO can be tuned via divalent substitution on the cation site to produce bilayers (or multilayers) of undoped ZnO (intrinsic ZnO (i-ZnO)) and lightly/highly/ultra-highly doped ZnO (n-ZnO) with successively increasing dopant concentration in order to magnify the static electric field and collect carriers efficiently. Substitution of Mg or Cd ions on the Zn cation sublattice enables band-gap tuning above and below the nominal 3.37 eV band-gap for the growth of heterostructures: Cd doping can decrease the band-gap to ~3.0 eV, whereas Mg doping can increase the band-gap to ~4.0 eV [35,36]. The intrinsic defect levels leading to n-type doping in nominally undoped ZnO are located approximately 0.01–0.05 eV below the conduction band and have been mostly attributed to Zn interstitials, oxygen vacancies, and process-induced hydrogen impurities. For ZnO, n-type conductivity is relatively easy to realize via excess Zn or doping with group-III elements (Al, Ga, and In) as Zn substituents. Group-VII elements (F, Cl, Br, and I) could also lead to high-quality n-type ZnO, as they occupy oxygen sites and contribute electrons [26]. On the contrary, p-type conductivity is difficult to achieve. Difficulty in achieving bipolar (n- and p-type) doping is a fairly common occurrence in wide band-gap semiconductors inclusive ZnSe and GaN [35]. Recent progress in acceptor doping of ZnO comprises three main approaches for generating p-type ZnO: substitution of group-IA elements (alkali metals) on a zinc site, co-doping of donors and acceptors, and substitution of group-VA elements on an oxygen site [26,36]. The relevant issues are [36]: sufficient incorporation of the appropriate dopant impurity species, impurities residing on the appropriate lattice site, and sufficiently small acceptor ionization energy to enable significant p-type conduction at room-temperature. Tremendous research efforts were focused on using group-IA (Li and Na), group-VA (N, P, As, Sb, and Bi), and group IB (Cu and Ag) elements or co-doped elements (Al–N, Ga–N, In–N, P–N, Zr–N, Li–N, P–Ga, N–As) as the p-type dopants [26]. Doping with group-VA elements resulted in proportion improvement of hole concentrations (10^16^–10^20^ cm^−3^) and resistivity values (10^−2^–10^2^ Ohm·cm) compared to group-IA (10^16^–10^19^ cm^−3^, 10–10^3^ Ohm·cm) [37]. The electrical conductivity of doped materials depends on both doping-induced defects and grain structure. Relating macroscopic electrical properties with the atomic structure is non-trivial because the derived materials are usually disordered and heterogeneous in nature.

Hence, with reference to intentional n-type doping of ZnO, the group-III elements were found to substitute for Zn, while the group-VII elements replaced O to contribute electrons. High electron concentrations (10^20^–10^21^ cm^−3^) and low resistivity (~10^−4^ Ohm·cm) have been achieved with Ga, Ga–In, Al, and Ga–Al dopants processed by vapor phase techniques, in particular molecular beam epitaxy (MBE), pulsed laser deposition (PLD), and rf-magnetron sputtering (RFMS) [26]. Similar applies to Al-doped ZnO films fabricated by atomic layer deposition (ALD) [38,39]. Changes in structural and electrical properties of ZnO due to Al doping studied using a quantum-chemical approach based on the Hartree–Fock method revealed electron density redistribution between different atomic orbitals within the same atom, leading to the generation of free electrons in the conduction band and increasing the n-type electrical conductivity and the band-gap width [40]. Precise analysis of defect type and effect on conductivity was reported for Al-doped ZnO (Al:ZnO (AZO)) with different doping levels and narrow particle-size distribution corroborated by quantum chemical calculations with an extended embedded cluster method at the DFT level [41]. Density Functional Theory (DFT) calculations revealed an increase of the optical band-gap and the electrical conductivity in Al:ZnO originated by an up-shift of the Fermi level [42]. Al-dopants induce, thereafter, Al-3s shallow donor states next to the Fermi level, at the conduction band minimum, assisting transitions. The current n-type doping techniques are well developed and ready to be used in various applications, for example, in the production of light emitting diodes (LEDs) and transparent Ohmic contacts.

The results can be significantly altered by introduction of growth techniques and precursor materials dependent on the quality-to-cost balance. B-doped ZnO (B:ZnO (BZO)) prepared by atomic layer deposition (ALD), at 150 °C, with triisopropyl borate ([(CH_3_)_2_CHO]_3_B (TIB)) source, exhibited resistivity values (3.5 × 10^−3^ Ohm·cm) similar to those of Al:ZnO processed with dimethylaluminium isopropoxide ((CH_3_)_2_AlOCH(CH_3_)_2_ (DMAI)) and lower than those of Al:ZnO (8 × 10^−3^ Ohm·cm) processed with trimethyl- aluminium (Al_2_(CH_3_)_6_ (TMA)) [43]. B:ZnO films prepared by plasma-enhanced chemical vapor deposition (PECVD) of diborane (B_2_H_6_) diluted in hydrogen (H_2_) gas, at 40.68 MHz and 180 °C, were used in mass production lines to obtain low-cost, large-area, transparent conductive oxide (TCO) contacts of a-Si:H/μc-Si:H solar cells [44]. Carrier concentrations were of the order 10^20^ cm^−3^ and resistivity varied in the range of (2.4–6.2) × 10^−3^ Ohm·cm (sheet resistance: 14.1–36.2 Ohm, thickness: 1700 nm). BZO has been considered as an ideal alternative for indium tin oxide (In_2_O_3_·SnO_2_ (ITO)) because of its low toxicity, natural abundance, and low price. Low-pressure chemical vapor deposited BZO films possess as-grown textured surfaces with pyramidal grains that can efficiently promote sunlight into the solar cells. On the contrary, ITO and AZO films deposited by magnetron sputtering are smooth and post-growth wet-chemical or ion etching is required to produce the desired surface finish.

Among the available deposition techniques, spray pyrolysis [45,46,47,48], as fast-rate thin-film growth procedure by pyrolysis techniques [49], is an effective chemical process for ZnO thin film growth by spraying a solution on a heated substrate; surface damage due to plasma is avoided, high vacuum is not required, and equipment costs are low. In- and Cu-doped ZnO films were deposited on glass substrates by spray pyrolysis at temperatures of 450–500 °C and doping levels of 2–3 at.% and 0, 2.5, 7.5 at.%, respectively [50,51]. Low resistivity of 4.0 × 10^−2^ Ohm·cm, lowered to 3.4 × 10^−3^ Ohm·cm by application of ultrasonic spray techniques [52], and carrier concentration of 7.0 × 10^19^ cm^−3^ were obtained for In:ZnO (IZO) [50]. The electrical resistivity (6.58, 5.84, 2.93 Ohm·cm) of Cu:ZnO (Cu: 0, 2.5, 7.5 at.%) decreased with dopant incorporation to values lower than those reported for DC and radio frequency (RF) magnetron-sputtered films [51], indicating that high-quality films can be obtained by inexpensive, easy-to-implement spray pyrolysis techniques. Zinc acetate [Zn(CH_3_COO)_2_·2H_2_O)] and copper chloride (CuCl_2_·2H_2_O) were used as precursors. Semiconductor doping with transition metals (Cu, Fe) has recently become of great importance. Cu:ZnO films with Cu up to 20 at.% (2, 5, 10, 15 and 20 at.%) have been deposited so far by ultrasonic spray pyrolysis (USP) [53]. For films with dopant concentrations < 15 at.%, the presence of a wurtzite-type phase was confirmed. Films with higher Cu content include a mixed oxide phase of Zn_x_Cu_1−x_O type. The (optical) band-gap energy of the films, deduced from the transmittance spectra, decreased gradually from 3.25 to 1.90 eV with the increase of Cu concentration. In the last decade, spray pyrolysis has also been applied to deposit ZnO films co-implanted/co-doped with rare-earth elements (Tb, Yb, Ce, Er, Dy). Zn_1−x_Yb_x_O (0 ≤ x ≤ 0.05) thin films, deposited on glass substrates from zinc chloride (ZnCl_2_) and ytterbium chloride (YbCl_3_·6H_2_O) solution, were n-type conducting with carrier density in the range 6.5 × 10^22^–1.4 × 10^21^ cm^−3^ and lowest electrical resistivity of 1.8 × 10^−2^ Ohm·cm for 1% Yb. The films exhibited 75–90% transmittance in the visible range, with a sharp absorption-onset at 375 nm, corresponding to the fundamental energy-gap of 3.3 eV [54]. Ytterbium/Terbium co-doped ZnO thin films exhibited highest carrier density of 2.3 × 10^21^ cm^−3^ and lowest electrical resistivity of 6.0 × 10^−3^ Ohm·cm. The (optical) band-gap energies of undoped ZnO and (Yb-Tb):ZnO, estimated from photoluminescence spectra, were 3.27 and 3.23–3.21 eV, respectively [55]. Band-gap shrinkage has commonly been observed on Al:ZnO, B:ZnO, and Cu:ZnO processed by hydrothermal techniques [56,57,58]. Liquid-phase processing of B:ZnO and Cu:ZnO by sol–gel techniques [59,60,61] resulted in carrier concentration and electrical resistivity limited to 10^15^ cm^−3^ and 10^2^ Ohm·cm, respectively.

Electrochemical deposition (ECD) techniques have similar prospects for application in material synthesis and large-volume production, yet distinctly superior rating for cost-effective overall processing of efficient chalcopyrite semiconductor based thin-film solar cells (CIS/CIGS TFSCs) extensively analyzed in our previous reports [1,2,3,4,5,6,7,8,9,10,11,12,13] of the List of References [1,2,3,4,5,6,7,8,9,10,11,12,13,14,15,16,17,18,19,20,21,22,23,24,25,26,27,28,29,30,31,32,33,34,35,36,37,38,39,40,41,42,43,44,45,46,47,48,49,50,51,52,53,54,55,56,57,58,59,60,61,62,63,64,65,66,67,68,69,70,71,72,73,74,75,76,77,78,79,80,81,82,83,84,85,86,87,88,89,90,91,92,93,94,95,96,97,98,99,100,101,102,103,104,105,106,107,108,109,110,111,112,113,114,115,116,117,118]. ECD is a reliable method for fabricating, with high reproducibility, excellent-quality n-type ZnO material with high carrier concentration, high carrier mobility, and low resistivity, as demonstrated in our previous reports [2,3] and the current study. Thin films of zinc-oxide highly to ultra-highly doped with aluminium (Al:ZnO) and indium (In:ZnO) dopants were processed from aqueous solution of zinc nitrate (Zn(NO_3_)_2_) and solute dopant concentrations of aluminium trichloride (AlCl_3_) and indium trichloride (InCl_3_) ranging up to 20 and 15 mM, respectively. Thus, scaling of the dopant concentration, in the solution, from the usually trimmed 3–4 mM of chlorides (InCl_3_ [62]) or sulfides (Al_2_(SO_4_)_3_ [63]) up to the ultra-high of 15–20 mM was utilized, in the frame of the present work, targeting to increase the internal field response and the carrier collection in CIS/CIGS TFSCs. Emphasis was given to the optical properties and the quantification of optical spectral distributions in order to calibrate the carrier concentration (cm^−3^) in the Al:ZnO and In:ZnO with respect to the solute dopant concentration of AlCl_3_ and InCl_3_ (mM) dissolved in the electrochemical solution. Carrier concentrations were deduced from optical reflectance spectra by rectifying the band-gap broadening in accordance with the Burstein–Moss effect and are compared to the results of sheet resistance measurements. High carrier concentrations in the Al:ZnO and In:ZnO window layer and front Ohmic contact of CIS/CIGS TFSCs are usually achieved by application of sputtering techniques under vacuum conditions. The achievement of adequately high carrier concentrations by liquid-phase techniques, specifically electrochemical deposition (ECD), was a central objective of the present study. The comparison and consistency of the optical characterization results with the results of electrical characterization was also a central point of interest that has carefully been addressed. The main aspects of the present scientific research can thus be summarized as follows:

**(1)** ECD processing of ZnO highly doped with aluminium (Al) and indium (In),

**(2)** Determination of carrier concentrations (carrier densities),

**(3)** Calibration of ECD solute dopant concentration with respect to carrier density,

**(4)** Compatibility of optical and electrical characterization results.

Aluminium-doped zinc-oxide (AZO) films represent a promising upcoming alternative to transparent conductive oxide (TCO) films mainly because of their excellent electrical and optical properties, cost-effective and abundant raw materials, non-toxic nature, long-term environmental stability, and facile fabrication. It is noted that calibration of the most frequently used AlCl_3_ solute dopant (mM) to atomic percent (at.%) incorporated in the Al:ZnO lattice was already accessed by scanning electron microscopy (SEM) combined with energy dispersive X-ray analysis (EDAX) in one of the author’s previous works [2], and correspondence of 20 mM AlCl_3_ to 12.5 at.% Al in AZO was reported. In accordance with the published literature, doping of electrochemically deposited Al:ZnO was generally constrained to 5 at.% Al^3+^ [64].

The use of non-polluting, cost-effective, moderate-temperature, large-area, fast-rate ECD techniques with large-scale performance in processing of absorber-, buffer-, window-layer and front Ohmic contact of CIS/CIGS TFSCs is expected to reduce costs, eliminate process incompatibilities, minimize number of process steps in roll-to-roll processes, and increase production rates.

## 2. Materials and Methods

Electrochemical deposition techniques have been applied in thin-film processing of intrinsic zinc-oxide (i-ZnO) and n-type conductive zinc-oxide (n-ZnO) doped with aluminium (Al:ZnO) or indium (In:ZnO). The aluminium (Al) and indium (In) were intentionally used for doping to rate the performance of low-cost Al versus high-quality In dopant and the replacement capability of the low-abundance In element. Both i-ZnO and n-ZnO films were deposited from aqueous solutions of 0.05 M zinc nitrate (Zn(NO_3_)_2_), pure or treated with metal chloride additives (AlCl_3_: 1–20 mM and InCl_3_: 5–15 mM) at 80 °C. Dependent on the dopant precursor concentration, for Al:ZnO processing, the pH of 0.05 M Zn(NO_3_)_2_ solution, with AlCl_3_ ranging from 7 to 20 mM, was in the range 3.69–3.32 at 25.1 °C. In case of highly diluted AlCl_3_ dopant of 1 mM, the solution pH was 4.15. Aqueous solutions of AlCl_3_ are known to be acidic, indicative of partial hydrolysis of the Al^3+^ ion: [Al(H_2_O)_6_]^3+^ ⇌ [Al(OH)(H_2_O)_5_]^2+^ + H^+^. Precipitation of Al(OH)_3_ or In(OH)_3_, as registered in Ref. [62], was not observed. Moreover, it is not expected within the short deposition time (300 s) and the high dopant concentrations. Lower and higher negative electrochemical potential E_C_ of −0.8 V and −1.2 V was applied for the deposition of i-ZnO and n-ZnO, respectively, in a standard three-electrode configuration with zinc (Zn) counter and saturated calomel electrode (SCE) reference powered by EG&G Princeton Applied Research 263A potentiostat/galvanostat, and a Witeg MSH-20D Hotplate Stirrer. ZnO formation on the cathode side using a zinc nitrate-based solution proceeds as follows [2,3]:

(1) $$Zn{\left(NO_{3}\right)}_{2}↔Zn^{2+}+2NO_{3}{}^{-}$$

(2) $$NO_{3}{}^{-}+H_{2}O+2e^{-}\to NO_{2}{}^{-}+2OH^{-}$$

(3) $$Zn^{2+}+2OH^{-}↔Zn{\left(OH\right)}_{2}$$

(4) $$Zn{\left(OH\right)}_{2}\to ZnO+H_{2}O$$

The film thickness was adjusted to the current-deposition time (I(t)) characteristics of the ECD process following Faraday’s law:

(5) $$d=\frac{j\;M\;t}{n\;F\rho }$$

with *j* current density (A/cm^2^), *M* molecular weight (g), *t* deposition time (s), ρ material density (g/cm^3^), *F* Faraday constant (*F* = 96,485.3365(21) C (≈96,500 C)), and *n* number of charge transferred. The actual thickness of the films was determined by scanning electron microscopy (SEM) on the cross-section of the Al:ZnO/ZnO/Mo/glass and In:ZnO/ZnO/ZnSe/Mo/glass film structures [2,3].

Both Al:ZnO and In:ZnO films were deposited by ECD with thickness ~400 nm (deposition time t_n-ZnO_ = 300 s) on ZnO templates: Al:ZnO was deposited on ~40 nm i-ZnO template (t_i-ZnO_ = 300 s) on Mo/glass substrate (1.0 × 3.0 cm^2^); In:ZnO was deposited on ~110 nm i-ZnO template (t_i-ZnO_ = 600 s) on ZnSe/Mo/glass substrate (1.5 × 1.5 cm^2^). The lattice mismatch between the hexagonal ZnO template (a = b = 3.251 Å) and the cubic Mo substrate (a = 3.146 Å) is ~3%. ZnSe is deposited with wurtzite structure (hexagonal ZnSe) on amorphous glass and polycrystalline substrates [4]. The lattice- and thermal- mismatch of the ZnO layer (a = 3.25 Å, α_th_ = 4.31 × 10^−6^ K^−1^) and the hexagonal ZnSe buffer (a = 3.98 Å, α_th_ = 7.8 × 10^−6^ K^−1^) is ~20%. However, the Al:ZnO/ZnO bilayer is deposited on intermediate ZnSe buffer of CIGS TFSCs almost strain-free, because part of the tensile mismatch strain to the underlying ZnSe is cancelled by the (hydrostatic) compressive strain (ε_h_) in Al:ZnO [3]. Residual stresses of the deposited layers result, in general, from differences in the lattice constants and the thermal expansion coefficients of substrate and epilayer. Hydrostatic strain effects evolve apparently by incorporation of Al (or In) dopant in the ZnO lattice. The ZnSe buffer is intentionally probed for ZnO deposition because it possesses wide band-gap (2.7 eV), lattice mismatch to ZnO (a(_ZnO_) = 3.25 Å, a(_ZnSe_) = 3.98 Å) lower than the CdS to ZnO mismatch (a(_ZnO_) = 3.25 Å, a(_CdS_) = 4.14 Å), and non-toxic elements, which makes it a very promising, environmental friendly material for replacement of the CdS buffer in CIGS TFSCs [65]. Both the ZnO template and the n-ZnO films (Al:ZnO, In:ZnO) are thus expected to be grown free of mismatch strain.

Both dopant elements (Al, In) incorporated in n-ZnO are highly conductive (Al: 3.8 × 10^7^ S/m and In: 1.2 × 10^7^ S/m). The atomic radius of the Al dopant (r(_Al_) = 1.82 Å), however, diverges from the atomic radius of Zn (r(_Zn_) = 1.53 Å) less than the In atomic radius (r(_In_) = 2.00 Å), indicating that Al can more easily be embedded in the ZnO lattice as substitute or interstitial [41] under assimilation of strain [2,3]. The dopant type is expected to modify the film microstructure: nanostructured In:ZnO and uniformly structured Al:ZnO films are thus produced by incorporation of 5 at.% In and Al [64], respectively, with the structural modifications amplified by the increase of dopant concentration and/or deposition time [2]. The band-gap of Al^3+^ (In^3+^) doped films is broadened with the increase of the aluminium (indium) dopant concentration [3,62]. The electrical conductivity of aluminium doped zinc-oxide materials (AZO) depends on doping-induced defects and grain structure. Al_Zn_ substitutes contribute with 1e− to the increase of local charge density. Al interstitials, tetrahedrally or octahedrally coordinated to O sites, offer already 3e− [41].

The Al:ZnO and In:ZnO films were subjected to post-deposition annealing at 300 °C, for 2 h, in an oven purged with argon gas and sealed at atmospheric overpressure to avoid defect formation by contamination with air and oxygen chemisorption at the surface and grain boundaries.

The ECD-processed n-ZnO/i-ZnO bilayers were structurally, optically, and electrically characterized by X-ray diffraction (XRD), scanning electron microscopy (SEM), UV-VIS-NIR spectrophotometry, and capacitance spectroscopy (C-V/I-V). ZnO characterization results were also reported in our previous publications [2,3]. The present study adopts the optical spectral measurements targeting quantification of carrier concentrations in the fabricated Al:ZnO and In:ZnO layers through exploitation of the band-gap broadening by the Burstein–Moss effect. Simultaneously, the calibration of carrier concentration in relation to the AlCl_3_ and InCl_3_ solute dopant concentration, dissolved in Zn(NO_3_)_2_ solution in multiple proportions ranging up to 20 and 15 mM, respectively, contributes essentially to the CIS/CIGS photovoltaic research and technology applications.

## 3. Results and Discussion

**Figure 1:**
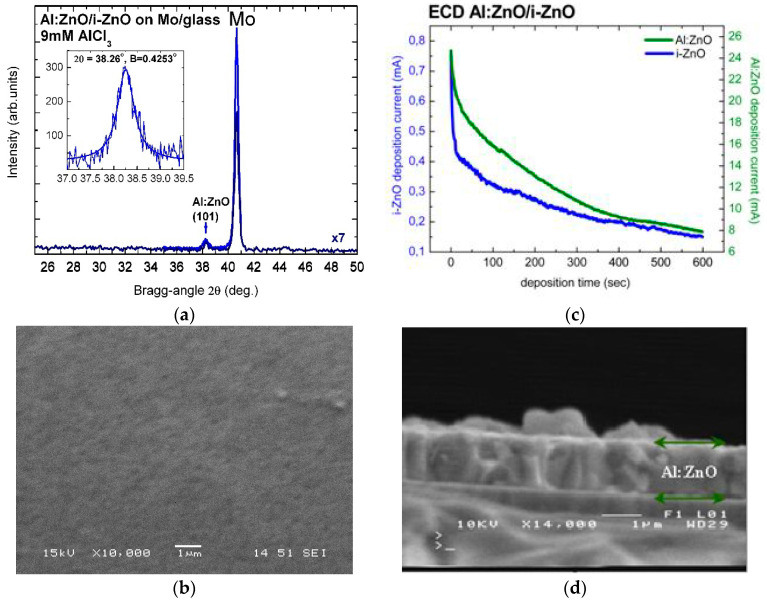
(**a**) High-resolution X-ray diffractogram and (**b**) SEM image of Al:ZnO layer deposited with t_Al:ZnO_ = 300 s on i-ZnO/Mo/glass substrate by ECD from solution with 9 mM AlCl_3_ (in the inset: fitting of (101) reflection peak (2θ = 38.26°, B = 0.4253°)). (**c**) I(t) characteristics of the ECD process (i-ZnO (blue line), Al:ZnO (green line)) and (**d**) SEM cross-sectional image of a thicker Al:ZnO layer on i-ZnO/Mo/glass.

The high crystalline quality of electrochemically deposited Al:ZnO (AZO) and In:ZnO (IZO) thin films was confirmed by X-ray diffractograms. Figure 1a includes a long-range (25–50°) low-resolution XRD scan of Al:ZnO on i-ZnO/Mo/glass, processed by ECD from solution with 9 mM AlCl_3_, and a high-resolution pattern of the Al:ZnO reflection from ($10\bar{1}1$) crystallographic plane at Bragg angle 2θ = 38.26° and Bragg width B = 0.4253°. The intense peak at 2θ = 40.66° is assigned to Bragg reflection from the (110) crystallographic plane of the molybdenum (Mo) substrate. Al:ZnO layers deposited by ECD on ZnO/Mo/glass, with deposition time t_Al:ZnO_ = 300 s and [AlCl_3_] = 9 mM, consist of regular fine grains, as demonstrated in the SEM image of Figure 1b. The average grain size of the polycrystalline film structure deduced from the measured width B (full width at half-peak maximum (FWHM) in radians) of the Al:ZnO diffraction peak by using the Scherrer formula (*D* = (*Kλ*)/(*Bcosθ*) with *K* = 0.9 (spherical crystallites) and *λ* = 1.5406 Å (Cu-Kα_1_ line) [66]) was D = 20 nm.

The thickness of i-ZnO and n-ZnO films (Figure 1c,d) was determined by the current-in-dependence-of-time I(t) characteristics of the ECD process and by SEM on the cross-section of the respective layers. Figure 1d is a SEM image of the cross-section of an Al:ZnO layer with deposition time 600 s and thickness 1.17 μm. The thickness can also be deduced from the current versus deposition-time characteristic curve (I(t)), in Figure 1c, by integration following exponential decay fit 2nd-order. For Al:ZnO/ZnO samples with 600 s deposition time and active area 2 cm^2^, Equation (5) (Faraday’s law) yields layer thickness of d(i-ZnO) = 280 nm and d(Al:ZnO) = 1350 nm. The charge density jt (C/cm^2^) is obtained as [current(I) × deposition time(t)]/deposit area(A) from the I(t) integration. The molecular weight M and material density ρ of ZnO are 81.38 g and 5.606 g/cm^3^, respectively, the charge transfer by formation of ZnO is n = 4, and Faraday’s constant F = 96,485.3365 C (≈96,500 C). The layer thickness of 1.35 μm calculated with Equation (5) from the I(t) curve of the deposition process is in agreement, within experimental and calculation errors, with the results of SEM imaging. ZnO layer thickness relevant for CIGS TFSC processing of front contact (n-ZnO/i-ZnO) with deposition times 300–400 s can, therefore, unambiguously be determined from the I(t) characteristics of the ECD process. Divergences between effective and calculated film thickness are usually due to local concentration gradients in the solution, gradual reduction of the solute concentrations with increasing deposition time, over- or underestimation of the deposited film area, and irreducible changes in molecular weight and material density of highly to ultra-highly doped ZnO (n-ZnO). The thickness of doped ZnO layers deposited with equal times as non-doped layers is considerably greater than that of the non-doped because of the significantly higher conductivity of the ECD bath as a result of the higher ion concentrations (Al^3+^, or alternately In^3+^, and Cl^3-^) in the presence of AlCl_3_ or InCl_3_ dopants.

Consequently, doping of ZnO with metal chlorides (AlCl_3_, InCl_3_) in Zn(NO_3_)_2_ solution at moderate temperature (80 °C) was proven to be versatile, well reproducible, nanocomposite-promoting, and reliable in view of Al(OH)_3_ and Al_2_O_3_ [67] precipitant suppression.

### 3.1. Reflectance Spectra of Al:ZnO and In:ZnO

#### 3.1.1. Determination of Band-Gap Energies by Application of the KUBELKA-MUNK Approximation and the TAUC Formalism

The reflectance spectra of Al:ZnO/ZnO and In:ZnO/ZnO bilayers were recorded in the NUV-VIS region from 180 to 400 nm.

**Figure 2:**
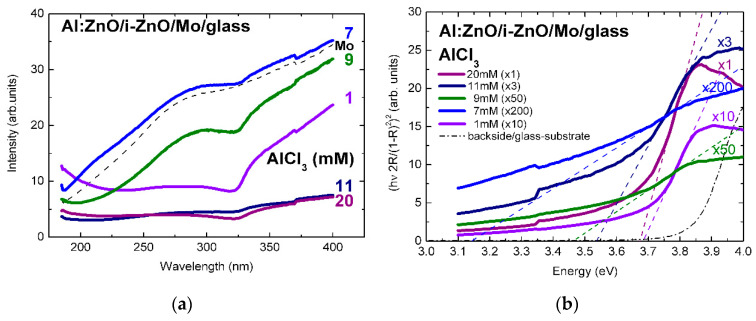
(**a**) Reflectance spectra of Al:ZnO (AZO) thin films deposited by ECD on Mo/glass substrates from Zn(NO_3_)_2_ solution with AlCl_3_ solute dopant concentration in the range 1–20 mM and (**b**) tauc-plots of Al:ZnO film reflectance in the Kubelka–Munk approximation [3].

The surface reflectivity of Al:ZnO/ZnO on Mo/glass, in Figure 2a, varies in the range 5–35% (normalized intensity) by varying the AlCl_3_ solute dopant concentration, in the ECD process, from 1 to 20 mM and reaches, at 7 mM, a maximum comparable with the reflectivity of the molybdenum back contact over the measured spectral range [3]. Further increase of the ECD precursor concentration, particularly above 10 mM, is followed by decrease of the spectral reflectance through increasing surface roughness, possibly because of (hydrostatic) strain-induced surface faceting up to coalescence breaking, as observed in Refs. [2,3]. Despite spatial variations up to 10% associated with local variations of surface roughness, curvature changes associated with the spectral reduction of reflectance through absorbance, at (optical) band-gap energy of Al:ZnO, are evidenced throughout the concentration changes of solute dopant successively increased from 1 mM to 7, 9, 11, and ultra-high 20 mM.

**Figure 3:**
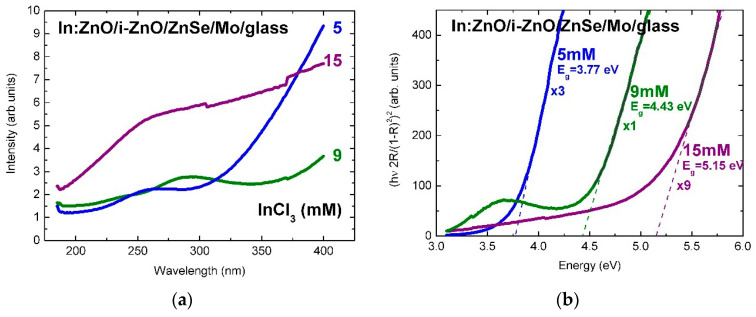
(**a**) Reflectance spectra of In:ZnO (IZO) thin films deposited by ECD on ZnSe/Mo/glass substrates from Zn(NO_3_)_2_ solution with InCl_3_ solute dopant concentration in the range 5–15 mM and (**b**) tauc-plots of the In:ZnO film reflectance in the Kubelka–Munk approximation.

The surface reflectivity of In:ZnO/ZnO on ZnSe/Mo/glass, in Figure 3a, is significantly lower than the reflectivity of Al:ZnO/ZnO. In analogy with the reflectivity of Al:ZnO/ZnO/Mo/glass, it is reduced from 9.5% to 3.5% (normalized intensity) with the increase of the InCl_3_ solute dopant concentration from 5 to 9 mM and reestablished at 15 mM, possibly due to predominance of doping over strain effects.

The (optical) band-gap of the Al:ZnO/ZnO and the In:ZnO/ZnO films was extracted from the reflectance spectra similarly to Refs. [2,3] by combining the Kubelka–Munk approximation [68,69,70] and the Tauc formalism [71] with the optical properties of semiconductor materials [72,73,74,75,76,77]. Despite the fact that the principal techniques of optical characterization are spectroscopic ellipsometry and modulation spectroscopy (photo- and electro-reflectance of derivative nature and spectral shape) applied in Refs. [2,4,7,9,10,11,12,13,33], the results of transmittance/reflectance measurements with the respective approximations refined in Refs. [78,79] have generally been proved highly useful. Moreover, the results of the quantification of the optical properties of ZnO-Nanorods (antireflective coating (ARC) of CIGS TFSCs) grown by ECD on ZnO templates from Zn(NO_3_)_2_ solution and characterized by photoreflectance (PR, E_g_(ZnO) = 3.32 eV) and transmittance (TR, E_g_(ZnO) = 3.35 eV) spectroscopy are in agreement (within experimental and calculation errors), as reported in a previous author’s study [2]. Similar applies to ZnSe (buffer layer of CIGS TFSCs) deposited by electron-beam evaporation (EBD) and characterized by PR (E_g_(ZnSe) = 2.69 eV) and ZnSe grown by chemical bath deposition (CBD) and characterized by TR (E_g_(ZnSe) = 2.73 eV) in the author’s study cited as Ref. [4].

The radiation resulting from the reflection, refraction, diffraction, and absorption by randomly oriented crystallites of polycrystalline materials is considered as diffuse (or volume) reflection (in contrast to regular (or directional) reflection from a plane phase boundary). Based on the absorption (*K*) and scattering (*S*) per unit layer thickness of the reflecting medium assumed to be a continuum, in the limiting case of an infinitely thick layer, the Kubelka–Munk theory provides an approximation useful at any wavelength:

(6) $$\frac{K}{S}=F(R_{\infty })=\frac{{\left(1-R_{\infty }\right)}^{2}}{2R_{\infty }}$$

where $R_{\infty }$ is the limiting reflectance (diffuse reflectance of a sample of infinite thickness) and *F*($R_{\infty }$) is usually termed the remission or Kubelka–Munk (K–M) function. The K–M approximation is thus applicable to layers thick enough to ensure that a further increase in thickness will fail to change the reflectance.

In the parabolic band approximation, band-gap energy *E_g_* and absorption coefficient α of a direct band-gap semiconductor are related through the general Equation [76]:

(7) $$\alpha h\nu =C{\left(h\nu -E_{g}\right)}^{1/2}$$

where α is the linear absorption coefficient of the material, *h*ν is the photon energy, and *C* is a proportionality constant. In most direct transition materials, an absorption tail to lower photon energies exists, which is described by the so-called Urbach rule [74,76]. Band tails are usually related to doping and phonon-assisted transitions.

Considering the K–M absorption coefficient *K* as proportional to α and the K–M scattering coefficient *S* as wavelength-independent constant, the sample absorption, in Equation (7), can be expressed in terms of the inverse remission function of Equation (6) as follows:

(8) $${\left({\left(F(R_{\infty })\right)}^{-1}\;h\nu \right)}^{2}=C^{\prime }\left(h\nu -E_{g}\right)$$

A plot of ${\left({\left(F(R_{\infty })\right)}^{-1}h\nu \right)}^{2}$ in dependence of energy (hν), with *F*($R_{\infty }$) calculated according to Equation (6), and linear fit up to the point of curve inflection gives, by intersection with the energy axis, the band-gap energy of the sample.

The linear fits of the absorption edges of the Al:ZnO epilayers are depicted in the tauc-plots of Figure 2b with the AlCl_3_ solute dopant concentration of 1–20 mM as parameter. The respective band-gap energies are in the range: *E_g_*(Al:ZnO) = 3.14–3.68 eV. The tauc-plot fits of the In:ZnO epilayers with the InCl_3_ solute dopant concentration in the range 5–15 mM are presented in Figure 3b with band-gap energies *E_g_*(In:ZnO): 3.77 eV (5 mM), 4.43 eV (9 mM), and 5.15 eV (15 mM). The experimental and calculation errors are estimated to ±0.05 eV.

#### 3.1.2. Calculation of Carrier Concentrations Based on the Burstein–Moss Effect

The density of carriers (carrier concentration) in non-degenerate semiconductors is related to the density of available states and the probability that each of these states is occupied.

The density of states for electrons in the conduction band (CB) and holes in the valence band (VB) is given by [80]:

(9) $$g_{CB}=\frac{8\pi \sqrt{2}}{h^{3}}m_{e}^{*3/2}\sqrt{E-E_{C}},\;E\geq E_{C}$$

(10) $$g_{VB}=\frac{8\pi \sqrt{2}}{h^{3}}m_{e}^{*3/2}\sqrt{E_{V}-E},\;E\leq E_{V}$$

where $E_{C}$, $E_{V}$ are the bottom of conduction and top of valence band, respectively, $m_{e}^{*}$ is the effective electron mass, and h= 6.626 × 10^−34^ J·s (=4.136 × 10^−15^ eV⋅s) is the Planck constant.

The density of electrons is obtained by integrating the product of the density of states and the electron probability distribution. Since electrons are Fermions, their probability function is the Fermi–Dirac function *f*(*E*) that is also called the Fermi function and applies to all particles with half-integer spin. The Fermi function converges to zero at higher energies. The upper integral limit can be, therefore, replaced by infinity. The carrier density at thermal equilibrium is thus given by [80]:

(11) $$N=\int_{E_{C}}^{\infty }g_{CB}f(E)dE$$

(12) $$f(E)=\frac{1}{1+e^{\left(E-E_{F}\right)/kT}}$$

where $E_{F}$ is the Fermi energy, k=1.381 × 10^−23^ J/K (=8.617 × 10^−5^ eV/K) the Boltzmann constant, and T the temperature with kT= 4.11 × 10^−21^ J (=25.7 meV) at 298 K.

Combining Equations (11) and (12) yields:

(13) $$N=\int_{E_{C}}^{\infty }\frac{8\pi \sqrt{2}}{h^{3}}m_{e}^{*3/2}\sqrt{E-E_{C}}\frac{1}{1+e^{\left(E-E_{F}\right)/kT}}dE$$

Non-degenerate semiconductors are defined as semiconductors for which the Fermi energy $E_{F}$ is at least 3kT away from either band edge. This definition allows the Fermi function *f*(*E*) to be replaced by the Maxwell–Boltzmann distribution function:

(14) $$N=\int_{E_{C}}^{\infty }\frac{8\pi \sqrt{2}}{h^{3}}m_{e}^{*3/2}\sqrt{E-E_{C}}\;e^{\left(E_{F}-E\right)/kT}dE$$

The carrier density integral can then be solved analytically, yielding:

(15) $$N=N_{C}\;e^{\left(E_{F}-E\right)/kT}\;,\;N_{C}=2{\left(\frac{2\pi m_{e}^{*}kT}{h^{2}}\right)}^{3/2}$$

with $N_{C}$ being the effective density of states in the conduction band.

The Fermi energy, $E_{F}$, is obtained from:

(16) $$E_{F}=E_{C}+kT\ln\frac{N}{N_{C}}$$

Equation (13) can be solved analytically at *T* = 0 K, since *f*(*E*) = 1, $E\leq E_{F}$ and *f*(*E*) = 0, $E>E_{F}$; therefore, it can be simplified to:

(17) $$N=\int_{E_{C}}^{{}^{E}F}g_{CB}(E)dE$$

and integration yields [80]:

(18) $$N=\frac{8\pi 2\sqrt{2}}{3h^{3}}m_{e}^{*3/2}{\left(E_{F}-E_{C}\right)}^{3/2}\;\mathrm{for}\;E_{F}\geq E_{C}$$

This expression can be used to approximate the carrier density in heavily degenerate semiconductors, provided that: $\left(E_{F}-E_{C}\right)\gg KT$ and $\left(E_{F}-E_{C}\right)>0$.

In a degenerate sample, the height of the Fermi level above the bottom of the conduction band, $E_{F}-E_{C}$, increases very rapidly with increasing electron density [81].

In the parabolic band approximation with spherical constant-energy surfaces (isotropic case), there is no variation in the effective mass with the crystal directions. Hence, for an electron at the bottom of the conduction band, with effective mass $m_{e}^{*}$, the relation between the energy *E* and wave vector *k* is simplified [82]:

(19) $$E=h^{2}k^{2}/2m_{e}^{*}\;,\;dE/dk=h^{2}k/m_{e}^{*}$$

The density of allowed states in *k*-space and energy interval Δ*E* is then straightforward:

(20) $$dn/dk=2\left(4\pi k^{2}\right)\;,\;dn/dE=8\pi m_{e}^{*}k/h^{2}=\left(8\pi /h^{3}\right){\left(2m_{e}^{*}{}^{3}E\right)}^{1/2}$$

Integrating gives the total number of states in an energy interval Δ*E* above the bottom of the conduction band [82]:

(21) $$N=\int_{0}^{\Delta E}dn=\frac{8\pi 2\sqrt{2}}{3h^{3}}m_{e}^{*3/2}{\left(\Delta E\right)}^{3/2}$$

**Figure 4:**
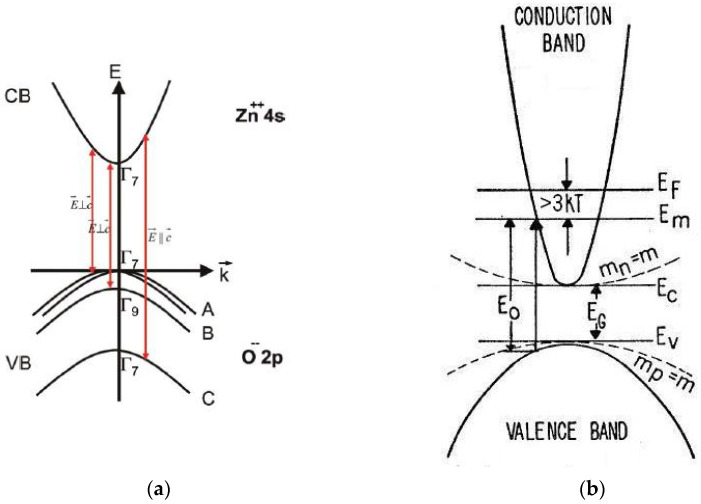
Schematic representation of: (**a**) the band structure of wurtzite-type (hexagonal) ZnO with valence band ordering at the Γ-point and splitting into three valence bands (A, B, C) originated by the crystal field and spin-orbit interaction (the polarizations $\vec{E}//\vec{c}$, $\vec{E}⊥\vec{c}$ of dipole-allowed band-to-band transitions are indicated) [25] and (**b**) conduction band ordering in case of an n-type sample exhibiting the Burstein–Moss effect [81] with $E_{F}$ the Fermi-level and $E_{m}$ the lowest unfilled level in the conduction band (the corresponding energy–momentum curves $E(\vec{k})$ for a semiconductor with electron and hole effective masses m_n_ = m_p_ = m are depicted by dotted lines).

The band structure of ZnO [25,83] is shown schematically in Figure 4a. ZnO is a wide, direct band-gap semiconductor that crystallizes in the wurtzite structure. The conduction band (CB) with symmetry $\Gamma_{7}$ including spin originates from the empty 4s states of Zn^++^ or the antibonding sp^3^ hybrid states in view of ionic or covalent binding, respectively. The valence band (VB) results from the occupied 2p states of O^–^ or the bonding sp^3^ states; it is split by the hexagonal crystal field and spin orbit interaction into three two-fold degenerate sub-bands named A, B and C. The usual ordering in wurtzite type semiconductors is: A $\left(\Gamma_{9}\right)$, B $\left(\Gamma_{7}\right)$ and C $\left(\Gamma_{7}\right)$. In ZnO, inverted VB ordering has been introduced [84]. The symmetry ($\Gamma_{9}$ or $\Gamma_{7}$ character) of the upper valence band (sub-band A) has been the subject of controversy for a long time. Based on the polarization properties of the free exciton transitions, most researchers assume that the symmetry of the A-valence sub-band is $\Gamma_{7}$ [25]. Additional evidence of the $\Gamma_{7}$ character of the upper valence band and the inverted VB ordering was provided by detailed optical studies of ionized donor-bound excitons and donor–acceptor pair recombination in bulk, n-type ZnO [85].

The band structure of semiconductors is significantly modified when a high concentration of impurities (dopants) is introduced into the lattice [86,87,88,89,90,91,92,93,94]. At sufficiently high concentrations, impurities may form a band that merges with one of the intrinsic bands of the semiconductor. For a random or disordered distribution of impurities, the density of states at the band edges forms tails into the energy gap. The band structure of a perfect crystal may thus be perturbed by the core potential of the specific impurity as well as by the localized strains (deformation potential) induced by a certain misfit of the impurity. As the density of impurities increases, the perturbations overlap and tend to shift the bands to lower energies. If the valence band is not shifted as strongly as the conduction band, the net effect is a shrinkage of the energy gap. The band-gap narrowing (BGN) due to exchange interactions of free carriers, electron-hole interaction (the correlation energy shift), and carrier-(ionized)impurity interaction has an important influence on the optical properties and device performance of semiconductors, and is expressed as [90,91,92,93]:

(22) $$\frac{\Delta E}{R_{e}}=\frac{1.83}{r_{s}}\frac{\Lambda }{N_{b}^{1/3}}+\frac{0.95}{r_{s}^{3/4}}+\frac{\pi }{2}\frac{1}{r_{s}^{3/2}N_{b}}\left(1+\frac{m_{\min}^{*}}{m_{maj}^{*}}\right)$$

$R_{e}$ is the effective Rydberg energy for a carrier bound to a dopant atom, and $r_{s}$ is the average distance $r_{a}$ between majority carriers normalized to the effective Bohr radius α:

(23) $$R_{e}=\frac{13.6m_{d}}{e^{2}}eV$$

(24) $$r_{s}=\frac{r_{a}}{\alpha }\;,\;r_{a}={\left(3/4\pi N\right)}^{1/3}\;,\;\alpha =\frac{0.53\varepsilon }{m_{d}}\times 10^{-8}\;\mathrm{cm}$$

N is the doping concentration, ε is the dielectric constant, and $m_{d}$ is the effective density of state-mass of the carriers in the majority band divided by the free electron mass. The semiconductor is assumed to be uncompensated and all impurities ionized so that N is also the concentration of free carriers. Λ is a correction factor that accounts for anisotropy of the bands, in n-type semiconductors, and for interaction between the heavy- and light-hole bands, in p-type semiconductors, $N_{b}$ is the number of equivalent band extrema, and $m_{maj}^{*}$ and $m_{\min}^{*}$ are majority- and minority-carrier density-of-state effective masses, respectively.

The Burstein–Moss effect [81], illustrated in Figure 4b, evolves in semiconductor materials with highly increasing doping. At particularly high dopant concentrations, an increase in the energy band-gap, defined as the energy separation between the top of the valence band and the unoccupied energy states in the conduction band, is observed. The shift to higher energy (blue shift) arises because the Fermi energy $E_{F}$ of heavily n-type doped semiconductors lies in the conduction band. The (optical) band-gap widening (BGW) with increasing carrier concentration is thus related to the rise of the Fermi level in the conduction band of a degenerate semiconductor. The optical transition of an electron from the valence band to the conduction band is vertical, meaning that the photon wave-vector (k=2π/λ) is small compared to the wave-vector of the electrons at the Fermi energy $\left(k_{F}={\left(3\pi^{2}\right)}^{1/3}\right)$. The optical absorption limit of a degenerate n-type semiconductor involves vertical transitions from the filled valence band to the lowest unfilled level $E_{m}$ in the conduction band, which lies below the Fermi level $E_{F}$ [81] (Figure 4b). The filled states block thermal or optical excitation. The optical energy gap $E_{O}$ determined by the onset of interband absorption is, therefore, given by the energy separation between $E_{m}$ and the corresponding level in the filled valence band, which, assuming spherical energy surfaces, lies $\left(m_{n}/m_{p}\right)\;E_{m}$ below the top of the filled valence band. In this case, the optical gap $E_{O}$ differs from the minimum separation between the bands $E_{G}$ [81]. Provided that the curvature and position of the bands are independent of doping and the effective masses of valence and conduction bands are known, the shift in energy (Equations (18) and (21)) can be used as an accurate and contactless method of determining the carrier concentrations in doped semiconductor samples [92,93,94].

In the case of IZO (In:ZnO) [94], for example, the incorporation of In into ZnO causes the In atom to be ionized into In^3+^, which then replaces the Zn^2+^ ion in the ZnO host lattice. This replacement contributes one free electron and thus increases the carrier concentration. The ionic radii of In and Zn are 0.080 and 0.074 nm, respectively. The incorporation of In into the ZnO host lattice is thus also expected to cause film stress. The In atom may otherwise reside in an interstitial position and become a neutral defect that does not contribute to the free carrier concentration. Another possibility is that the In interstitials act as donors and contribute three electrons to the carrier concentration: $In^{0}\to In^{3+}+3e^{-}$. In the band filling model, the lowest states in the conduction band are already filled with electrons, as the carrier concentration is further increased to accommodate the conduction band density of states, $N_{C}$, given by Equation (15): $N_{C}=2{\left(2\pi m_{e}^{*}kT\right)}^{3/2}/h^{3}$ [80,94]. The value of $N_{C}$ is, thereafter, dependent on the effective electron mass $m_{e}^{*}$. In ZnO, with $m_{e}^{*}$ = 0.24$m_{0}$ ($m_{0}$ = 9.109 × 10^−31^ kg) [85], evaluation of Equation (15) results in $N_{C}$ = 2.92 × 10^18^ cm^−3^. This implies that the optical band-gap increases for carrier concentrations higher than ≈3 × 10^18^ cm^−3^.

Consequently, the increase in carrier concentration in degenerate semiconductors may cause two opposite effects:

band-gap narrowing (BGN) and band-gap widening (BGW) [92,93].

**Figure 5:**
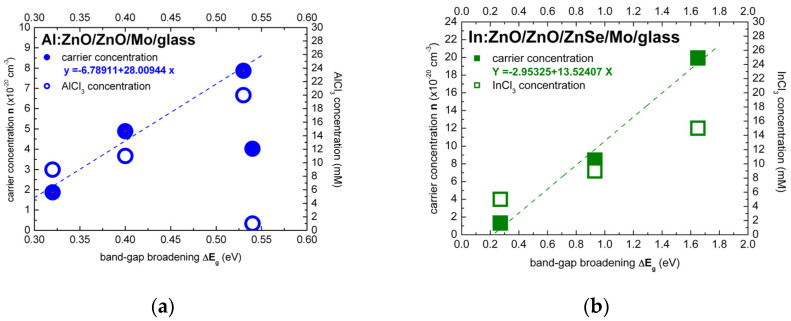
Carrier concentration in dependence of the energy band-gap broadening of ECD deposited thin films of highly doped: (**a**) Al:ZnO on ZnO/Mo/glass and (**b**) In:ZnO on ZnO/ZnSe/Mo/glass calculated under consideration of the Burstein–Moss effect (dashed lines are guide to the eye).

The Burstein–Moss effect was adapted, in the present study, in order to quantify the band-gap widening (BGW) and extract the carrier densities of highly to ultra-highly doped ZnO thin films with aluminium (Al) and indium (In) dopants. The carrier concentrations of ECD deposited Al:ZnO on ZnO/Mo/glass and In:ZnO on ZnO/ZnSe/Mo/glass by varying the AlCl_3_ (1, 7, 9, 11, 20 mM) and the InCl_3_ (5, 9, 15 mM) solute dopant concentration in the Zn(NO_3_)_2_ solution were evaluated using Equation (21) (equivalent, in this regard, to Equation (18)). The evaluation results of ZnO doping with Al and In dopants are presented in Figure 5a,b, respectively, and also inserted in Table 1 (Al:ZnO) and Table 2 (In:ZnO) comprising optical and electrical properties.

The distribution of gap energies is a result of the competition between two reciprocal interdependent effects: band-gap narrowing (BGN) through increase of donor-level population followed by band-gap widening (BGW) through over-increase of donor-level population and up-shift of the Fermi level, in the conduction band of a degenerate semiconductor, with intermediate properties between semiconductor and metal. The values of the (optical) energy gap $E_{g}$ were determined from the plots of ${\left(h\nu \;2R/{\left(1-R\right)}^{2}\right)}^{2}$ versus hν (Equations (6)–(8), Figure 2b and Figure 3b) and are inserted in Table 1 and Table 2. It should be noted that, as the dopant concentration and thus the carrier density increases, the film resistivity decreases and the optical band edge shifts to higher energies (lower wavelengths). This variation is typically a Burstein–Moss shift, which may be used to calculate the intrinsic band-gap $E_{0}$ in Al:ZnO and In:ZnO films as [95]:

(25) $$E_{g}=E_{0}+\Delta E$$

with ΔE being the distance of the Fermi level from the bottom of the conduction band. The Burstein–Moss shift ΔE may thus be expressed as:

(26) $$\Delta E=h^{2}E_{b}/8\pi^{2/3}\;,\;E_{b}=N^{2/3}/m_{e}^{*}$$

Equation (25) may then be rewritten to yield the (optical) energy gap:

(27) $$E_{g}=E_{0}+h^{2}N^{2/3}/8m_{e}^{*}\pi^{2/3}$$

Equation (27) suggests that a plot of $E_{g}$ versus $N^{2/3}$ should exhibit a linear variation. Assuming the effective mass $m_{e}^{*}$ to be independent of doping concentration, the intrinsic band-gap $E_{0}$ and the average value of $m_{e}^{*}$ can be determined from the intercept and slope, respectively, of this plot. Combining the optical ($E_{g}$) and electrical (N) measurement results, a value of the intrinsic ZnO (i-ZnO) band-gap $E_{0}$ = 3.12 eV was determined, which coincides, within experimental and calculation errors, with the band energy reference $E_{0}$ = 3.14 eV used in the following. The (average) value of the effective electron mass in Al:ZnO was $m_{e}^{*}$ = 0.67$m_{0}$.

Several structural models have been proposed to interpret the Al doping-induced disorder and categorize the Al defects. Only point defects have been considered so far, although a much wider variety of defects, such as edge and screw dislocations or planar and volume defects, seem to be possible. An overview of the suggested defects in Kröger–Vink notation [96] is given in [41]. Aluminium substituting for zinc (Al_Zn_) is considered to be the most probable defect impinging the material band-gap properties. Neutral defects involving three Zn^2+^ cations replaced by two Al^3+^ cations and a compensation cationic vacancy and acting as electron traps have also been considered [97]. Recently published micro-Raman, X-ray photoelectron spectroscopy (XPS), and spectroscopic ellipsometry (SE) analyses of Al:ZnO films prepared by co-sputtering of ZnO and Al at moderate temperatures (RT-188 °C) [98] indicate that the incorporated host atoms are Al^3+^ species in Zn^2+^ substitutional position and their amount increases following a direct monotonic trend with the deposition temperature.

For liquid-phase processing of Al:ZnO/ZnO by ECD, at 80 °C, the dependence of band-gap energy on AlCl_3_ solute dopant concentration and the respective atomic percent (at.%) of aluminium incorporated in the ZnO lattice, as determined by energy dispersive analysis X-ray (EDAX), are included in our previous reports [2,3]. For solute dopant concentrations below the 10 mM “threshold” detected in [2], introduction of Al^3+^ dopant inside the ZnO lattice is difficult from natural coordination preference [41,97,98] and consequently leads to a very low solubility limit (<1 at.%). The effect of low Al content of Al:ZnO is apparently defect-related band-gap narrowing from Eg = 3.68 to 3.14 eV (1–7 mM AlCl_3_). With the increase of AlCl_3_ concentration in the ECD solution, the atomic fraction of Al in the ZnO lattice is successively increased, the doping levels merge into dopant bands and take over in the vicinity of the 10 mM “threshold” with one order of magnitude higher Al content (>10 at.%). Evaluation of carrier concentrations with Equation (21): N = $\left(\left(8\pi 2\sqrt{2}\right)/3h^{3}\right)m_{e}^{*3/2}{\left(\Delta E\right)}^{3/2}$, $\Delta E=E_{Al:ZnO}-E_{0}$ has, therefore, band-energy reference $E_{0}=$ 3.14 eV corresponding to 7 mM AlCl_3_ solute dopant. It is worth to be noted that undoped ZnO films deposited from solution containing nitrate precursors by ECD [99] and hydrothermal [58] techniques exhibited band-gap energies of 3.1 and 3.2 eV, respectively. The dependence of the electron effective mass on the Al-dopant concentration (Al content in at.%) [100] has been also taken into account: $m_{e}^{*}$ = 0.37$m_{0}$ (Al: 0.36–0.66 at.%, AlCl_3_: 1–9 mM), $m_{e}^{*}$ = 0.57$m_{0}$ (Al: 11.39 at.%, AlCl_3_: 11 mM), $m_{e}^{*}$ = 0.59$m_{0}$ (Al: 12.53 at.%, AlCl_3_: 20 mM). The carrier densities varied in the range (1.88–7.87) × 10^20^ cm^−3^ for AlCl_3_ solute dopant concentrations between 9 and 20 mM (Figure 5a). Carrier concentrations of the order of 10^20^ cm^−3^ have thus been accessed by ECD processing of Al:ZnO thin films with Al-dopant incorporation in the order of 10 at.%.

The band-gap energy shift of In:ZnO on ZnO/ZnSe/Mo/glass was evaluated in analogy to the Al:ZnO shift. The band-energy reference $E_{0}=$ 3.50 eV was determined in a previous author’s report analyzing, among others, the optical properties of ZnO deposited by ECD on ZnSe/Cu [2]. The electron effective mass was set equal to $m_{e}^{*}$ = 0.35$m_{0}$ reported in Ref. [95]. The carrier densities increased from 1.32 × 10^20^ cm^−3^ to 1.99 × 10^21^ cm^−3^ with the increase of InCl_3_ solute dopant concentration from 5 to 15 mM (Figure 5b). The analysis results, in Table 2, indicate that doping of ECD-deposited ZnO with In to fabricate In:ZnO thin films proceeds effectively and carrier concentrations of the order of 10^20^ cm^−3^ are already attained at InCl_3_ solute dopant concentration of 5 mM.

#### 3.1.3. Calibration of the Dependence of the Al:ZnO and In:ZnO Carrier Concentration on the AlCl_3_ and InCl_3_ Solute Dopant Concentration

**Figure 6:**
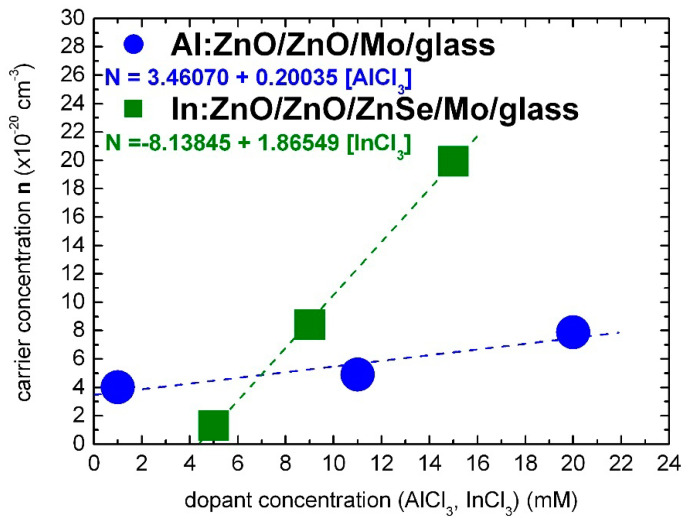
Carrier concentration of electrochemically deposited highly doped Al:ZnO (AZO) and In:ZnO (IZO) thin films in dependence, respectively, of the AlCl_3_ and InCl_3_ solute dopant concentration in the Zn(NO_3_)_2_ solution.

The optical characterization results of the present study lead to the conclusion that Al and In dopants can be incorporated in the ZnO lattice with similar effectiveness for solute dopant concentrations up to 7 mM of AlCl_3_ and InCl_3_, respectively, as demonstrated in Figure 6.

The carrier concentration of Al:ZnO appears to have reached a saturation limit of the order 10^20^ cm^−3^ at AlCl_3_ solute dopant concentration in the order of 10 mM. On the contrary, the carrier concentration of In:ZnO exhibits an upward trend and reaches 10^21^ cm^−3^ at InCl_3_ solute dopant concentration of 10 mM. The higher expenses of doping with indium are thus counterbalanced by the superior impact of a lower amount of the indium dopant.

Band-gap widening (Band-gap narrowing) unfolds also under the influence of compressive (tensile) stress generated by the incorporated donors [2,3]. The atomic and ionic radii of Al (1.82 Å, Al^3+^: 0.54 Å) and In (2.00 Å, In^3+^: 0.80 Å) diverge from the Zn radii (1.53 Å, Zn^2+^: 0.74 Å). Al and In dopants are embedded in the ZnO lattice as substitutes or interstitials under renormalization of strain. The lowest AlCl_3_ dopant concentration of 1 mM, in the present study, exceeds by one order of magnitude the Al(NO_3_)_3_ solute dopant concentrations of 0.1–0.3 mM, in Ref. [58], that lead distinctly to BGN. With the increase of dopant concentration from 0.1 to 1 mM, BGN is followed by BGW. At dopant concentrations of 1–10 mM, opposite BGN and BGW may result from the simultaneous or consecutive actions of energy–momentum and strain–stress overwhelmed for concentrations ≥10 mM by the BGW impinging energy–momentum dynamic. For the calibration of carrier concentration in dependence of solute dopant concentration, only the measurements primarily ([AlCl_3_] = 1 mM) or directly ([AlCl_3_] ≥ 10 mM) influenced by the Burstein–Moss effect were taken into account (Figure 6).

In the summary: the carrier densities $N_{\mathrm{Al}:\mathrm{ZnO}}$ and $N_{\mathrm{In}:\mathrm{ZnO}}$ of electrochemically deposited Al:ZnO (AZO) and In:ZnO (IZO) layers, deduced from optical spectra by evaluation of the Burstein–Moss shift, depend linearly on the solute dopant concentrations of [AlCl_3_] and [InCl_3_] in the Zn(NO_3_)_2_ solution. The calibration equations are given by:

(28) $$N_{\mathrm{Al}:\mathrm{ZnO}}\;{(\mathrm{cm}}^{-3})=(3.46+0.20\;{[\mathrm{AlCl}}_{3}]\;\mathrm{mM})\;{\times 10}^{20}$$

(29) $$N_{\mathrm{In}:\mathrm{ZnO}}\;{(\mathrm{cm}}^{-3})=(-8.14+1.87\;{[\mathrm{InCl}}_{3}]\;\mathrm{mM})\;{\times 10}^{20}$$

It has been observed that the Burstein–Moss effect plays an important role in the optical properties of tellurium (Te) compensated Ga_1−x_In_x_Sb bulk crystals, grown by the vertical Bridgman method, for alloy compositions greater than x = 0.5 and net donor concentrations in the 2.93 × 10^17^ to 2.63 × 10^18^ cm^−3^ range [101]. The III-V ternary alloy system GaSb–InSb with a band-gap in the range of 0.73–0.17 eV, at 300 K, is a promising candidate for high efficiency thermophotovoltaic cells operating in conjunction with low-temperature black body sources.

The Burstein–Moss effect has also been quantified in Co^2+^ doped Cu_2_GeSe_3_ single crystals, with band-gap energy of 0.85 eV, grown by the modified Bridgman technique to fabricate energetically broadened Cu_2_GeSe_3_:Co^2+^, and was found to be originated by the overlapping of the conduction band of the Cu_2_GeSe_3_ single crystal and the $T_{d}$ energy levels of the Co^2+^ ion of 0.932 and 0.825 eV at 298 K [102].

### 3.2. Resistivity of Al:ZnO and In:ZnO Characterized by Optical and Electrical Techniques

Earth-abundant Al:ZnO (AZO) is the most preferable transparent conductive oxide (TCO) to be used in optoelectronic and photovoltaic devices. TCOs are wide-band-gap semiconductor materials ($E_{g}$ > 3 eV) with high electrical conductivity (σ≈ 10^3^–10^4^ S/cm) and high optical transmittance (>85%) in the VIS-NIR spectral region. The large scale industrial use of standard TCO, indium tin oxide (ITO: In_2_O_3_·SnO_2_), is being reconsidered because of the low natural abundance and high cost of indium (In). The relatively low carrier mobility of AZO μ≈15.21 ± 0.04 cm^2^/V·s [103] is approached as a challenge to enhance the electrical conductivity $\sigma =Nq_{e}\mu$ ($q_{e}$ = 1.602 × 10^−19^ C) without compromising the optical transmittance. High electrical conductivity can be achieved by increasing the electron concentration N and maximizing the electron mobility μ. However, carrier mobility, in polycrystalline films, is restricted to values of 10–40 cm^2^/(V·s) decreasing with the increase of doping level because of enhanced scattering by ionized donors and their coupling with structural defects. In AZO, the Hall mobility was found to decrease from 16.2 to 6.2 cm^2^/V·s with the increase of the Al dopant concentration from 0.5 to 7 at.% [100]. Besides, high electron concentrations lead to increased IR absorption and can diminish the efficiency of solar cells. In CIGS thin-film solar cells (TFSCs), it is customary to use a high-to-low resistivity ρ=1/σ grading of the ZnO layer: an undoped layer of ZnO with higher resistivity is initially deposited on the buffer layer, followed by deposition of a highly doped low-resistive layer [3]. ECD allows convenient doping and thus resistivity grading by varying the solute dopant concentration.

The resistivity of ECD-deposited Al:ZnO and In:ZnO films deduced from optical measurements by varying the AlCl_3_ and InCl_3_ solute dopant concentration and thereby the electron density $N_{Al:ZnO}$ and $N_{In:ZnO}$, respectively, was calculated according to:

(30) $$\rho =\frac{1}{\sigma }=\frac{1}{Nq_{e}\mu (N)}\;,\;R=\rho \frac{l}{A}$$

with the carrier concentration N= $N_{Al:ZnO}$, $N_{In:ZnO}$, the elementary charge $q_{e}$ = 1.602 × 10^−19^ C, and μ(N) the electron density dependent electron drift mobility. The latter was extracted from Ref. [100] (AZO, averaged μ≈ 15.23 cm^2^/V·s) and Ref. [104] (IZO, 9 mM InCl_3_, 10.6 at.% In, μ= 2.41 cm^2^/V·s and 15 mM InCl_3_, 23.7 at.% In, μ= 1.32 cm^2^/V·s). The (electrical) resistance R, in Equation (30), depends on resistivity ρ, length l, and cross-sectional area A. The sheet resistance $R_{sheet}$ of a film with thickness d can be determined as [44,105]:

(31) $$R_{sheet}(N,d)=\frac{1}{dNq_{e}\mu (N)}$$

Given that the electron mobility μ(N) depends on the electron density N, the variance of $R_{sheet}$, at constant layer thickness d, is dominated by its dependence on electron density N.

**Figure 7:**
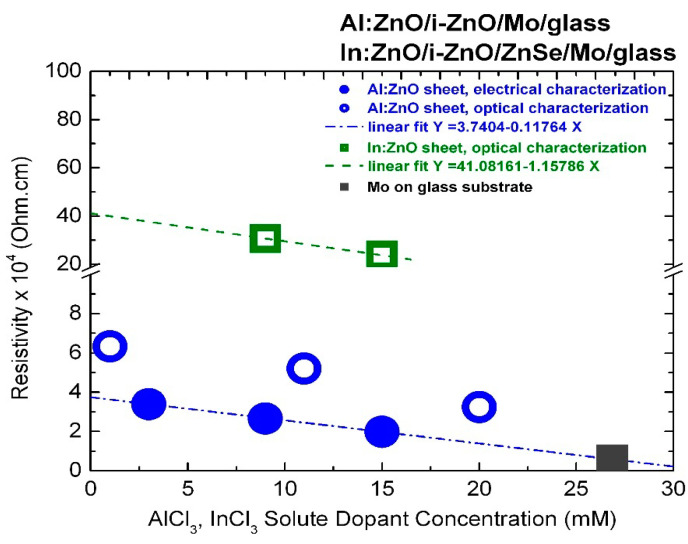
Resistivity of ECD deposited thin films of highly doped Al:ZnO on ZnO/Mo/glass determined by electrical (full circles) and optical (open circles) characterization techniques and In:ZnO on ZnSe/ZnO/Mo/glass determined by optical techniques (open squares) in comparison with the resistivity of the molybdenum (Mo) metallic film (full square).

The resistivity values of ECD-deposited Al:ZnO on ZnO/Mo/glass and In:ZnO on ZnO/ZnSe/Mo/glass thin films quantified by optical characterization techniques with respect to carrier concentrations determined from the upshift of the energy band-gap by the Burstein–Moss effect are presented in Figure 7 and inserted in Table 1 (Al:ZnO) and Table 2 (In:ZnO). The AZO resistivity in the order of 10^−4^ Ohm·cm is one order of magnitude lower than the resistivity of IZO in the order of 10^−3^ Ohm·cm and comparable with the resistivity of ITO of 5.1 × 10^−4^ Ohm·cm [105]. A resistivity of 3.0 × 10^−3^ Ohm·cm has also been reported for IZO films deposited by spray-pyrolysis techniques [106]. Compilation of literature data on the electrical properties of ZnO films prepared by various deposition techniques and doped with the group IIIB elements B, Al, Ga, and In shows that resistivity lower than 10^−3^ Ohm·cm can be achieved [107], whereby the lowest one is obtained with Al dopants [107,108].

The AZO and IZO resistivity determined by optical characterization techniques is most likely related to the transparent layer sequence of Al:ZnO/ZnO and In:ZnO/ZnO/ZnSe. It is possibly overestimated, since high-resistive undoped transparent layers (ZnO, ZnSe) are also involved. Sheet resistance measurements of In-pellet contacted highly doped Al:ZnO sheets deposited by ECD on ZnO/Mo/glass from Zn(NO_3_)_2_ solution with AlCl_3_ solute dopant in the range 3–15 mM [3] yielded resistivity values in the order of 10^−4^ Ohm·cm, as demonstrated in Figure 7.

The divergence Δρ(Ohm·cm) between the resistivity values of the Al:ZnO film determined by optical and electrical characterization techniques (Δρ= 2.9 × 10^−4^ Ohm·cm at 1 mM, with $\rho_{optical}=$ 6.5 × 10^−4^ Ohm·cm, $\rho_{electrical}=$ 3.6 × 10^−4^ Ohm·cm) is lowered (Δρ = 2.0 × 10^−4^ Ohm·cm at 20 mM, $\rho_{optical}=$ 3.4 × 10^−4^ Ohm·cm, $\rho_{electrical}=$ 1.4 × 10^−4^ Ohm·cm) with the increase of the AlCl_3_ dopant concentration and the corresponding increase of carrier density. The resistivity of the Al:ZnO sheet processed with 9 mM AlCl_3_ was $\rho_{\mathrm{Al}:\mathrm{ZnO}}$ = (2.7 ± 0.2) × 10^−4^ Ohm·cm. The sheet resistance of 400 nm thick molybdenum on glass substrate (Mo/glass) used to process the Al:ZnO/ZnO samples by ECD and also used as standard back contact of CIGS TFSCs was found to be of the same order: $\rho_{Mo}$ = 0.58 × 10^−4^ Ohm·cm, and is inserted in Figure 7 for comparison reasons. This value is exactly one order of magnitude higher than the molybdenum bulk resistance known from the literature: $\rho_{Mo}$ = 0.58 × 10^−5^ Ohm·cm at 300 K [109]. Sputtered molybdenum films with thickness of 0.5 μm and good adhesion to soda lime glass for CIGS device technology usually exhibit resistivity values by factor 5–10 higher than the resistivity of the bulk [110,111]. Hence, an ultra-heavily doped Al:ZnO front contact of a CIGS TFSC deposited from ECD solution with 27 mM AlCl_3_ is expected to be equally conductive to the molybdenum back contact.

### 3.3. I(V) Characteristics of Au/In:ZnO/ZnO/ZnSe/CIGS/Mo/Glass and c-Si Junctions

**Figure 8:**
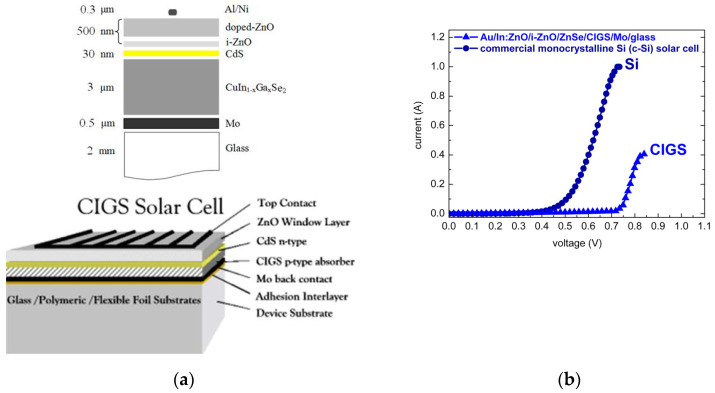
(**a**) Schematic cross-sectional representations of CIGS thin film solar cells with CIGS-absorber/CdS-buffer/ZnO-window on Mo/glass (alternately polymeric or flexible metal foil) and (**b**) comparison of chalcopyrite absorber based Au/In:ZnO/ZnO/ZnSe/CIGS/Mo/glass and commercial monocrystalline silicon (c-Si) current-voltage (I(V)) characteristics.

Chalcopyrite absorber-based photovoltaic technology [112] currently demonstrates the highest cell and module efficiencies of all inorganic thin-film technologies. Thin films of ternary and quaternary chalcopyrites (CuInSe_2_, CuInS_2_, CuGaSe_2_, CuGaS_2_, Cu(In,Ga)Se_2_) are easily prepared in a wide variety of compositions with high absorption coefficients α ≈ 10^5^ cm^−1^. The chalcopyrite sulfide CuInS_2_ (**CIS**), with an energy band-gap of 1.5 eV coinciding with the maximum of the solar energy spectrum, matches best the requirements for solar energy conversion. Its selenide counterpart CuInSe_2_ (**CISe**) with 1.0 eV energy gap, however, has proven to be a leading candidate for photovoltaic technology applications. For efficient chalcopyrite based thin film solar cells, a common practice has evolved with gallium (Ga) being added to obtain a CuIn_1−x_Ga_x_Se_2_ ([Ga]/([In] + [Ga]) ≤ 30%) quaternary alloy with a broader energy gap. Commercial Cu(In,Ga)Se_2_ (**CIGS**) thin-film solar cells (TFSCs) are realized as heterojunctions composed of a CIGSe absorber, a very thin CdS buffer layer (≤30 nm), and a ZnO window layer (Al:ZnO/ZnO), as illustrated in Figure 8a. Record efficiencies of Cu(In,Ga)Se_2_ solar cells have been prolonged beyond 20% [113] in the past decade. For Cu(In,Ga)Se_2_ and Cu(In,Ga)(Se,S)_2_ TFSCs, highest record efficiencies of 22.3–22.9% [114,115,116] have been achieved. A record efficiency of 23.35% has also been reported for Cd-free Cu(In,Ga)(Se,S)_2_ TFSCs with relatively complex Zn(O,S,OH)_x_/Zn_0.8_Mg_0.2_O double-buffer layers [117].

A main goal in CIGS photovoltaic technology is still the unification of processes applied to the growth of chalcopyrite absorber, buffer, and window layer. In commercial CIS/CIGS photovoltaics, the absorber is commonly grown by physical vapor deposition (PVD) specified as co-evaporation of the precursor materials at elevated temperatures (500–700 °C), the CdS buffer is grown by chemical bath deposition (CBD) at moderate temperatures (50 °C), and the ZnO window and front contact, usually a bilayer of lightly and highly doped ZnO, is sputtered at higher temperatures (500 °C). Switching between vacuum and non-vacuum techniques, dry- and wet-processing, moderate and elevated temperatures is rather complicated since it includes an increased number of process-steps and undesirable side-effects based on gas- or liquid-phase reactions and elemental interdiffusion at the interfaces. Moreover, CBD, despite low-cost precursor materials and simplicity, is less controllable than PVD. Processing in the liquid phase can otherwise sufficiently be managed by electrochemical deposition (ECD) techniques. In view of the efficiency-to-cost balance, the successive replacement of n- and p-type layers of CIGS TFSCs with layers processed by inexpensive chemical (CBD) and electrochemical (ECD) techniques has unambiguously great prospects. Moreover, the replacement of the toxic CdS buffer layer by environmental friendly and band alignment-favoring ZnSe is feasible [4,20]. In addition, ECD-processed antireflective coatings (ARCs) based on ZnO-Nanorods are compatible with the underlying n-ZnO/i-ZnO layers and suitable for surface finishing [2] without the prerequisites of surface polishing and texturing.

Comparative studies of ZnSe growth by electron beam-assisted PVD and by CBD were reported in a recent author’s publication [4]. The handling of low-cost, exclusively inorganic precursor compounds of low toxicity and the simplicity of the ECD process implementation under growth control maintenance initiated the growth of CuInSe_2_ and Cu(In,Ga)Se_2_ chalcopyrite absorbers [1] and n-ZnO/i-ZnO window layers [2,3] by ECD. The current scientific investigation is focused on ECD processing, optical characterization, and ECD parameter calibration of ZnO highly to ultra-highly doped with aluminium (Al:ZnO) and indium (In:ZnO) in order to maintain carrier densities achieved by thin film growth under vacuum conditions. Actualized carrier densities of n-type Ga-doped ZnO by an area-selective commercial focused ion beam (FIB) system were limited to 10^20^ cm^−3^ [118]. Among the aforementioned benefits of cost-efficiency, process-control, and moderate-temperature, ECD grants large-area deposition by sufficiently fast deposition rates, which are both essential steps towards monolithic CIS/CIGS photovoltaic module manufacture, and waste management through recycling.

The calibration of carrier densities with respect to solute dopant concentrations was an important task in order to methodize and generalize the ECD procedure for overall processing of CIS/CIGS TFSCs. Targeting overall wet processing and process engineering of CIS/CIGS TFSCs to overcome process incompatibilities and reduce production costs, CBD ZnSe buffer- and ECD In-ZnO/i-ZnO window-layer were deposited on PVD-grown Cu(In,Ga)Se_2_ chalcopyrite absorber. Heterojunctions of ECD ZnO/CBD ZnSe/PVD CIGS were fabricated. Following optimization of layer thickness and dopant concentration, the p–n junctions of Au/In:ZnO/i-ZnO/ZnSe/CIGS/Mo/glass exhibited I(V) characteristics competing with those of commercial crystalline silicon (c-Si) solar cells, as demonstrated in Figure 8b.

## 4. Conclusions

The carrier densities of highly to ultra-highly doped Al:ZnO and In:ZnO thin films electrochemically deposited (ECD) from zinc nitrate (Zn(NO_3_)_2_) solutions with AlCl_3_ and InCl_3_ solute dopant concentrations in the range 1–20 mM and 5–15 mM, respectively, at negative electrochemical potential of E_C_ = −1.2 V and moderate temperature of 80 °C, were quantified by optical characterization techniques and evaluation of the Burstein–Moss shift. The carrier densities $N_{Al:ZnO}$ and $N_{In:ZnO}$ vary linearly with the solute dopant concentration, have equal amounts of 5 × 10^20^ cm^−3^ at 7 mM solute dopant, and tend to saturation in Al:ZnO, with 7.5 × 10^20^ cm^−3^ at 20 mM, and maximization in In:ZnO, with 2 × 10^21^ cm^−3^ at 15 mM. The ZnO doping rates deduced from the optical spectra of Al:ZnO (AZO) and In:ZnO (IZO) films exhibiting the Burstein–Moss effect are: $0.2\;\times 10^{20}{\mathrm{cm}}^{-3}/\mathrm{mM}[AlCl_{3}]$ for 1 mM ≤ $\left[AlCl_{3}\right]$ ≤ 20 mM and $2\;\times 10^{20}{\mathrm{cm}}^{-3}/\mathrm{mM}[InCl_{3}]$ for 5 mM ≤ $\left[InCl_{3}\right]$ ≤ 15 mM. The rate of zinc-oxide doping with indium is thus an order of magnitude higher than the rate of doping with aluminium. 

The resistivity of the Al:ZnO and In:ZnO films calculated with carrier densities extracted from optical measurements was in the order of 10^−4^ and 10^−3^ Ohm·cm, respectively, in agreement with values known from the literature. The determination of carrier densities by optical spectroscopy and the verification of the consistency of optical and electrical characterization results confirmed the quantification of semiconductor doping processes by optical techniques.

Cu(In,Ga)Se_2_ chalcopyrite semiconductor based heterojunctions with In:ZnO/ZnO window-layer and front-contact deposited by ECD on the ZnSe/Cu(In,Ga)Se_2_ absorber/buffer layer sequence on Mo/glass substrate exhibited I(V) characteristics competing with monocrystalline silicon (c-Si) I-Vs.

## Figures and Tables

**Table 1:** **Optical and Electrical Properties of Al:ZnO (AZO) thin films:**$\left[AlCl_{3}\right]$ solute dopant concentration, Al dopant amount in Al:ZnO, band-gap energy $E_{g}$, electron density $N_{Al:ZnO}$, and resistivity $\rho_{optical}$, $\rho_{electrical}$.

Sample	[AlCl_3_] mM	Al at.%	E_g_ eV	N_Al:ZnO_ × 10^20^ cm^−3^	ρ _Optical_ × 10^−4^ Ohm·cm	ρ _Electrical_ × 10^−4^ Ohm·cm
**Al:ZnO on ZnO/Mo/glass**	1	0.36	3.68	4.03	6.34	
	3					3.39
	7	0.52	3.14			
	9	0.66	3.46	1.88		2.66
	11	11.39	3.54	4.89	5.21	
	15					1.98
	20	12.53	3.57	7.87	3.24	

**Table 2:** **Optical and Electrical Properties of In:ZnO (IZO) thin films:**$\left[InCl_{3}\right]$ solute dopant concentration, In dopant amount in In:ZnO, band-gap energy $E_{g}$, electron density $N_{In:ZnO}$, and resistivity $\rho_{optical}$.

Sample	[InCl_3_] mM	In at.%	E_g_ eV	N_In:ZnO_ × 10^20^ cm^−3^	ρ _Optical_ × 10^−4^ Ohm·cm
ZnO on ZnSe/Cu *	0		3.5		
**In:ZnO on ZnO/ZnSe/Mo/glass**	5		3.77	1.32	
	9	10.58	4.43	8.43	30.66
	15	23.69	5.15	19.93	23.71

* Ref. [2].

## References

[1] Papadimitriou D., Roupakas G., Sáez-Araoz R., Lux-Steiner M.-C., Nickel N.H., Alamé S., Vogt P., Kneissl M. (2015). Quality CuInSe_2_ and Cu(In,Ga)Se_2_ thin films processed by single-step electrochemical deposition techniques. Mater. Res. Express.

[2] Papadimitriou D.N. (2016). Structural, optical, electrical properties, and strain/stress of electrochemically deposited highly doped ZnO layers and nanostructured ZnO antireflective coatings for cost-effective photovoltaic device technology. Thin Solid Films.

[3] Papadimitriou D.N., Roupakas G., Roumeliotis G.G., Vogt P., Köhler T. (2016). Optimization of Electrochemically Deposited Highly Doped ZnO Bilayers on Ga-Rich Chalcopyrite Selenide for Cost-Effective Photovoltaic Device Technology. Energies.

[4] Papadimitriou D.N. (2018). Vacuum and Liquid-Phase Processing of ZnSe Buffer-Layer for Chalcopyrite Absorber Based Photovoltaic Technology. ECS J. Solid State Sci. Technol..

[5] Xue C., Papadimitriou D., Raptis Y.S., Esser N., Richter W., Siebentritt S., Lux-Steiner M.C. (2003). Compositional dependence of Raman scattering and photoluminescence emission in Cu_x_Ga_y_Se_2_ thin films. J. Appl. Phys..

[6] Xue C., Papadimitriou D., Esser N. (2004). Mapping of gradient composition Cu_x_Ga_y_Se_2_ film properties using Raman and PL-spectroscopy. J. Phys. D Appl. Phys..

[7] Xue C., Papadimitriou D., Esser N. (2004). Optical characterization of epitaxial Cu_x_Ga_y_Se_2_-layers by photoreflectance spectroscopy. Thin Solid Films.

[8] Papadimitriou D., Esser N., Xue C. (2005). Structural properties of chalcopyrite thin films studied by Raman spectroscopy. Phys. Status Sol..

[9] Theodoropoulou S., Papadimitriou D., Rega N., Siebentritt S., Lux-Steiner M.-C. (2006). Raman and photoreflectance study of CuIn_1−x_Ga_x_Se_2_ epitaxial layers. Thin Solid Films.

[10] Xu H.-Y., Papadimitriou D., Zoumpoulakis L., Simitzis J., Lux-Steiner M.-C. (2008). Compositional and temperature dependence of the energy band gap of Cu_x_In_y_Se_2_ epitaxial layers. J. Phys. D Appl. Phys..

[11] Theodoropoulou S., Papadimitriou D., Anestou K., Cobet C., Esser N. (2009). Optical properties of CuIn_1−x_Ga_x_Se_2_ quaternary alloys for solar-energy conversion. Semicond. Sci. Technol..

[12] Anestou K., Papadimitriou D. (2012). Optical modulation techniques applied in the analysis of chalcopyrite semiconductor heterostructures. J. Phys. D Appl. Phys..

[13] Papadimitriou D. (2015). Application of optical spectroscopic techniques in the characterization of elastic strain effects in semiconductor heterostructures and nanostructures and in semiconductor-based thin-film solar cells. Phys. Status Solidi B.

[14] Caballero R., Kaufmann C.A., Efimova V., Rissom T., Hoffmann V., Schock H.-W. (2013). Investigation of Cu(In,Ga)Se_2_ thin-film formation during the multi-stage co-evaporation process. Prog. Photovolt. Res. Appl..

[15] Mainz R., Weber A., Rodriguez-Alvarez H., Levcenko S., Klaus M., Pistor P., Klenk R., Schock H.-W. (2015). Time-resolved investigation of Cu(In,Ga)Se_2_ growth and Ga gradient formation during fast selenization of metallic precursors. Prog. Photovolt. Res. Appl..

[16] Rau U., Schmidt M. (2001). Electronic properties of ZnO/CdS/Cu(In,Ga)Se_2_ solar cells-aspects of heterojunction formation. Thin Solid Films.

[17] Schulmeyer T., Kniese R., Hunger R., Jaegermann W., Powalla M., Klein A. (2004). Influence of Cu(In,Ga)Se_2_ band gap on the valence band offset with CdS. Thin Solid Films.

[18] Chopra K.L., Paulson P.D., Dutta V. (2004). Thin-Film Solar Cells: An Overview. Prog. Photovolt. Res. Appl..

[19] Hariskos D., Spiering S., Powalla M. (2005). Buffer layers in Cu(In,Ga)Se_2_ solar cells and modules. Thin Solid Films.

[20] Hofmann A., Pettenkofer C. (2011). Surface orientation dependent band alignment for CuInSe_2_–ZnSe–ZnO. Appl. Phys. Lett..

[21] Ellmer K., Klein A., Ellmer K., Klein A., Rech B. (2007). ZnO and Its Applications. Transparent Conductive Zinc Oxide: Basics and Applications in Thin Film Solar Cells.

[22] Chen J., Aé L., Aichele C., Lux-Steiner M.-C. (2008). High internal quantum efficiency ZnO nanorods prepared at low temperature. Appl. Phys. Lett..

[23] Janotti A., Van de Walle C.G. (2009). Fundamentals of zinc oxide as a semiconductor. Rep. Prog. Phys..

[24] Klingshirn C., Hauschild R., Priller H., Decker M., Zeller J. (2005). ZnO rediscovered—Once again!?. Superlattices Microstruct..

[25] Klingshirn C., Fallert J., Zhou H., Sartor J., Thiele C., Maier-Flaig F., Schneider D., Kalt H. (2010). 65 years of ZnO research—Old and very recent results. Phys. Status Solidi B.

[26] Xiu F., Xu J., Joshi P.C., Bridges C.A., Paranthaman M.P., Paranthaman M.P., Wong-Ng W., Bhattacharya R.N. (2016). ZnO Doping and Defect Engineering—A Review. Semiconductor Materials for Solar Photovoltaic Cells.

[27] Wang J., Isshiki M., Kasap S., Capper P. (2006). Wide-Bandgap II-VI Semiconductors: Growth and Properties. Springer Handbook of Electronic and Photonic Materials.

[28] Özgür Ü., Alivov Y.I., Liu C., Teke A., Reshchikov M.A., Doğan S., Avrutin V., Cho S.-J., Morkoç H. (2005). A comprehensive review of ZnO materials and devices. J. Appl. Phys..

[29] Morkoç H., Özgür Ü. (2009). Zinc Oxide: Fundamentals, Materials and Device Technology.

[30] Adachi S. (2005). Properties of Group-IV, III-V and II-VI Semiconductors.

[31] Adachi S., Kasap S., Capper P. (2006). III-V Ternary and Quaternary Compounds. Springer Handbook of Electronic and Photonic Materials.

[32] Hanada T., Yao T., Hong S.-K. (2009). Basic Properties of ZnO, GaN, and Related Materials. Oxide and Nitride Semiconductors: Processing, Properties, and Applications.

[33] Papadimitriou D.N. (2020). Calibration of Polarization Fields and Electro-Optical Response of Group-III Nitride Based c-Plane Quantum-Well Heterostructures by Application of Electro-Modulation Techniques. Appl. Sci..

[34] Dietl T., Ohno H., Matsukura F., Cibert J., Ferrand D. (2000). Zener model description of ferromagnetism in zinc-blende magnetic semiconductors. Science.

[35] Norton D.P., Heo Y.W., Ivill M.P., Ip K., Pearton S.J., Chisholm M.F., Steiner T. (2004). ZnO: Growth, doping & processing. Mater. Today.

[36] Reynolds J.G., Reynolds C.L. (2014). Progress in ZnO Acceptor Doping: What Is the Best Strategy?. Adv. Condens. Matter Phys..

[37] Tang K., Gu S.-L., Ye J.-D., Zhu S.-M., Zhang R., Zheng Y.-D. (2017). Recent progress of the native defects and p-type doping of zinc oxide. Chin. Phys. B.

[38] Dhakal T., Nandur A.S., Christian R., Vasekar P., Desu S., Westgate C., Koukis D.I., Arenas D.J., Tanner D.B. (2012). Transmittance from visible to mid infra-red in AZO films grown by atomic layer deposition system. Sol. Energy.

[39] Li Y., Yao R., Wang H., Wu X., Wu J., Wu X., Qin W. (2017). Enhanced Performance in Al-Doped ZnO Based Transparent Flexible Transparent Thin-Film Transistors Due to Oxygen Vacancy in ZnO Film with Zn–Al–O Interfaces Fabricated by Atomic Layer Deposition. ACS Appl. Mater. Interfaces.

[40] Maldonado F., Stashans A. (2010). Al-doped ZnO: Electronic, electrical and structural properties. J. Phys. Chem. Solids.

[41] Avadhut Y.S., Weber J., Hammarberg E., Feldmann C., Schmedt-auf-der-Günne J. (2012). Structural investigation of aluminium doped ZnO nanoparticles by solid-state NMR spectroscopy. Phys. Chem. Chem. Phys..

[42] Fan Q., Yang J., Yu Y., Zhang J., Cao J. (2015). Electronic Structure and Optical Properties of Al-doped ZnO from Hybrid Functional Calculations. Chem. Eng. Trans..

[43] Garcia-Alonso D., Potts S.E., van Helvoirt C.A.A., Verheijen M.A., Kessels W.M.M. (2015). Atomic layer deposition of B-doped ZnO using triisopropyl borate as the boron precursor and comparison with Al-doped ZnO. J. Mater. Chem. C.

[44] Li W., Du J., Tang L., Tian Y., Xue F., Jiang Q., Pan S. (2020). Influence of boron doping amount on properties of ZnO:B films grown by LPCVD technique and its correlation to a-Si:H/μc-Si:H tandem solar cells. J. Mater. Sci. Mater. Electron..

[45] Aranovich J., Ortiz A., Bube R.H. (1979). Optical and electrical properties of ZnO films prepared by spray pyrolysis for solar cell applications. J. Vac. Sci. Technol..

[46] Paraguay F.D., Estrada W.L., Acosta D.R.N., Andrade E., Miki-Yoshidac M. (1999). Growth, structure and optical characterization of high quality ZnO thin films obtained by spray pyrolysis. Thin Solid Films.

[47] Ayouchi R., Leinen D., Martın F., Gabas M., Dalchiele E., Ramos-Barrado J.R. (2003). Preparation and characterization of transparent ZnO thin films obtained by spray pyrolysis. Thin Solid Films.

[48] Ashour A., Kaid M.A., El-Sayed N.Z., Ibrahim A.A. (2006). Physical properties of ZnO thin films deposited by spray pyrolysis technique. Appl. Surf. Sci..

[49] Bayan E.M., Petrov V.V., Volkova M.G., Yu Storozhenko V., Chernyshev A.V. (2021). SnO_2_–ZnO nanocomposite thin films: The influence of structure, composition and crystallinity on optical and electrophysical properties. J. Adv. Dielectr..

[50] Yoshino K., Oyama S., Kato M., Oshima M., Yoneta M., Ikari T. (2008). Annealing effects of In-doped ZnO films grown by spray pyrolysis method. J. Phys. Conf. Ser..

[51] Licurgo J.S.C., de Almeida Neto G.R., Paes Junior H.R. (2020). Structural, electrical and optical properties of copper-doped zinc oxide films deposited by spray pyrolysis. Cerâmica.

[52] Biswal R., Maldonado A., Vega-Pérez J., Acosta D.R., De La Luz Olvera M. (2014). Indium Doped Zinc Oxide Thin Films Deposited by Ultrasonic Chemical Spray Technique, Starting from Zinc Acetylacetonate and Indium Chloride. Materials.

[53] Roguai S., Djelloul A. (2020). Structural and optical properties of Cu-doped ZnO films prepared by spray pyrolysis. Appl. Phys. A.

[54] Soumahoro I., Schmerber G., Douayar A., Colis S., Abd-Lefdil M., Hassanain N., Berrada A., Muller D., Slaoui A., Rinnert H. (2011). Structural, optical, and electrical properties of Yb-doped ZnO thin films prepared by spray pyrolysis method. J. Appl. Phys..

[55] El hat A., Chaki I., Essajai R., Mzerd A., Schmerber G., Regragui M., Belayachi A., Sekkat Z., Dinia A., Slaoui A. (2020). Growth and Characterization of (Tb,Yb) Co-Doping Sprayed ZnO Thin Films. Crystals.

[56] Yu Q., Li J., Li H., Wang Q., Cheng S., Li L. (2012). Fabrication, structure, and photocatalytic activities of boron-doped ZnO nanorods hydrothermally grown on CVD diamond film. Chem. Phys. Lett..

[57] Thakur S.H., Sharma N., Varkia A., Kumar J. (2014). Structural and optical properties of copper doped ZnO nanoparticles and thin films. Adv. Appl. Sci. Res..

[58] Shah A., Ahmad M., Rahmanuddin, Khan S.H., Aziz U., Ali Z., Khan A., Mahmood A. (2019). The role of Al doping on ZnO nanowire evolution and optical band gap tuning. Appl. Phys. A.

[59] Bojorge C.D., Cánepa H.R., Gilabert U.E., Silva D., Dalchiele E.A., Marotti R.E. (2007). Synthesis and optical characterization of ZnO and ZnO:Al nanocrystalline films obtained by the sol-gel dip-coating process. J. Mater. Sci. Mater. Electron..

[60] Tsayn C.-Y., Hsu W.-T. (2013). Sol–gel derived undoped and boron-doped ZnO semiconductor thin films: Preparation and characterization. Ceram. Int..

[61] Joshi B.C., Chaudhri A.K. (2022). Sol−Gel-Derived Cu-Doped ZnO Thin Films for Optoelectronic Applications. ACS Omega.

[62] Machado G., Guerra D.N., Leinen D., Ramos-Barrado J.R., Marotti R.E., Dalchiele E.A. (2005). Indium doped zinc oxide thin films obtained by electrodeposition. Thin Solid Films.

[63] Lovchinov K., Ganchev M., Rachkova A., Nichev H., Angelov O., Mikli V., Dimova-Malinovska D. (2012). Structural and optical properties of electrochemically deposited ZnO films in electrolyte containing Al_2_(SO_4_)_3_. J. Phys. Conf. Ser..

[64] Chu D., Li S. (2012). Growth and Electrical Properties of Doped ZnO by Electrochemical Deposition. New J. Glass Ceram..

[65] Papadimitriou D., Roupakas G., Chatzitheodoridis E., Halambalakis G., Tselepis S., Sáez-Araoz R., Lux-Steiner M.C., Nickel N.H., Alamé S., Vogt P. Chemical and Electrochemical Processing of High Quality CIS/CIGS Absorber, Buffer, Window, and Anti-Reflective Coating for Low Cost Photovoltaic Technology. Proceedings of the 29th European Photovoltaic Solar Energy Conference and Exhibition, PVSEC.

[66] Cullity B.D. (1978). Elements of X-ray Diffraction.

[67] Souza A.D.V., Arruda C.C., Fernandes L., Antunes M.L.P., Kiyohara P.K., Salomão R. (2015). Characterization of aluminum hydroxide (Al(OH)_3_) for use as a porogenic agent in castable ceramics. J. Eur. Ceram. Soc..

[68] Torrent J., Barrón V. (2002). Diffuse Reflectance Spectroscopy of Iron Oxides. Encyclopedia of Surface and Colloid Science.

[69] Escobedo Morales A., Sánchez Mora E., Pal U. (2007). Use of diffuse reflectance spectroscopy for optical characterization of un-supported nanostructures. Rev. Mex. Fís..

[70] Sandoval C., Kim A.D. (2014). Deriving Kubelka–Munk theory from radiative transport. J. Opt. Soc. Am. A.

[71] Tauc J., Grigorovici R., Vancu A. (1966). Optical Properties and Electronic Structure of Amorphous Germanium. Phys. Stat. Sol..

[72] Wooten F. (1972). Optical Properties of Solids.

[73] Moss T.S., Burrell G.J., Ellis B. (1973). Semiconductor Opto-Electronics.

[74] Amirtharaj P.M., Seiler D.G. (1994). Optical Properties of Semiconductors. Handbook of Optics Volume II Devices Measurements and Properties.

[75] Grolik B., Kopp J. (2003). Optical Properties of Thin Semiconductor Films. Academia.edu.

[76] Mistrik J., Kasap S., Ruda H.E., Koughia C., Singh J., Kasap S., Capper P. (2006). Optical Properties of Electronic Materials: Fundamentals and Characterization. Springer Handbook of Electronic and Photonic Materials.

[77] Costa J.C.S., Taveira R.J.S., Lima C.F.R.A.C., Mendes A., Santos L.M.N.B.F. (2016). Optical band gaps of organic semiconductor materials. Opt. Mater..

[78] Souri D., Tahan Z.E. (2015). A new method for the determination of optical band gap and the nature of optical transitions in semiconductors. Appl. Phys. B.

[79] Dolgonos A., Mason T.O., Poeppelmeier K.R. (2016). Direct optical band gap measurement in polycrystalline semiconductors: A critical look at the Tauc method. J. Solid State Chem..

[80] Van Zeghbroeck B. (2011). Principles of Semiconductor Devices, Chapter 2: Semiconductor Fundamentals, 2.6. Carrier Densities. http://ecee.colorado.edu/~bart/book/book/chapter2/ch2_6.htm.

[81] Burstein E. (1954). Anoma1ous Optical Absorption Limit in InSb. Phys. Rev..

[82] Moss T.S. (1954). The Interpretation of the Properties of Indium Antimonide. Proc. Phys. Soc. B.

[83] Klingshirn C.F., Meyer B.K., Waag A., Hoffmann A., Geurts J. (2010). Zinc Oxide: From Fundamental Properties towards Novel Applications.

[84] Hopfield J.J. (1960). Fine structure in the optical absorption edge of anisotropic crystals. J. Phys. Chem. Solids.

[85] Meyer B.K., Alves H., Hofmann D.M., Kriegseis W., Forster D., Bertram F., Christen J., Hoffmann A., Straßburg M., Dworzak M. (2004). Bound exciton and donor-acceptor pair recombinations in ZnO. Phys. Stat. Sol. (B).

[86] Pankove J.I. (1971). Optical Processes in Semiconductors.

[87] Pankove J.I., Aigrain P. (1962). Optical Absorption of Arsenic-Doped Degenerate Germanium. Phys. Rev..

[88] Fistul’ V.I. (1969). Optical Properties of Heavily Doped Semiconductors. Heavily Doped Semiconductors. Monographs in Semiconductor Physics.

[89] Basu P.K. (2003). Theory of Optical Processes in Semiconductors: Bulk and Microstructures.

[90] Jain S.C., McGregor J.M., Roulston D.J. (1990). Band-gap narrowing in novel III-V semiconductors. J. Appl. Phys..

[91] Jain S.C., Roulston D.J. (1991). A simple expression for band gap narrowing (BGN) in heavily doped Si, Ge, GaAs and Ge_x_Si_1−x_ strained layers. Solid-State Electron..

[92] Kim C.E., Moon P., Kim S., Myoung J.-M., Jang H.W., Bang J., Yun I. (2010). Effect of carrier concentration on optical bandgap shift in ZnO:Ga thin films. Thin Solid Films.

[93] Ziabari A.A., Rozati S.M. (2012). Carrier transport and bandgap shift in n-type degenerate ZnO thin films: The effect of band edge nonparabolicity. Phys. B.

[94] Saw K.G., Aznan N.M., Yam F.K., Ng S.S., Pung S.Y. (2015). New Insights on the Burstein-Moss Shift and Band Gap Narrowing in Indium-Doped Zinc Oxide Thin Films. PLoS ONE.

[95] Sarkar A., Ghosh S., Chaudhuri S., Pal A.K. (1991). Studies on Electron Transport Properties and the Burstein-Moss Shift in Indium-doped ZnO Films. Thin Solid Films.

[96] Kröger F.A. (1964). The Chemistry of Imperfect Crystals.

[97] Serier H., Gaudon M., Ménétrier M. (2009). Al-doped ZnO powdered materials: Al solubility limit and IR absorption properties. Solid State Sci..

[98] Sanzaro S., la Magna A., Smecca E., Mannino G., Pellegrino G., Fazio E., Neri F., Alberti A. (2016). Controlled Al^3+^ Incorporation in the ZnO Lattice at 188 °C by Soft Reactive Co-Sputtering for Transparent Conductive Oxides. Energies.

[99] Chevva H., Palla S., Sankaranarayanan S. (2015). Characterization and Properties Evaluation of Conducting Al-doped ZnO at low temperature by ECD Method. Orient. J. Chem..

[100] Romanyuk V., Dmitruk N., Karpyna V., Lashkarev G., Popovych V., Dranchuk M., Pietruszka R., Godlewski M., Dovbeshko G., Timofeeva I. (2016). Optical and Electrical Properties of Highly Doped ZnO:Al Films Deposited by Atomic Layer Deposition on Si Substrates in Visible and Near Infrared Region. Acta Phys. Pol. A.

[101] Pino R., Ko Y., Dutta P.S. (2004). Burstein-Moss shift in impurity-compensated bulk Ga_1−x_In_x_Sb substrates. J. Appl. Phys..

[102] Lee J.J., Yang C.S., Park Y.S., Kim K.H. (1999). The Burstein-Moss effect in Cu_2_GeSe_3_:Co^2+^ single crystals. J. Appl. Phys..

[103] Kumar N., Chowdhury A.H., Bahrami B., Khan M.R., Qiao Q., Kumar M. (2020). Origin of enhanced carrier mobility and electrical conductivity in seed-layer assisted sputtered grown Al doped ZnO thin films. Thin Solid Films.

[104] Pati S., Banerji P., Majumder S.B. (2015). Properties of indium doped nanocrystalline ZnO thin films and their enhanced gas sensing performance. RSC Adv..

[105] Chen Z., Zhuo Y., Tu W., Li Z., Ma X., Pei Y., Wang G. (2018). High mobility indium tin oxide thin film and its application at infrared wavelengths: Model and experiment. Opt. Express.

[106] Lee C., Lim K., Song J. (1996). Highly textured ZnO thin films doped with indium prepared by the pyrosol method. Sol. Energy Mater. Sol. Cells.

[107] Schmidt-Mende L., MacManus-Driscoll J.L. (2007). ZnO—Nanostructures, defects, and devices. Mater. Today.

[108] Ellmer K., Bikowski A. (2016). Intrinsic and extrinsic doping of ZnO and ZnO alloys. J. Phys. D Appl. Phys..

[109] Desal P.D., Chu T.K., James H.M., Ho C.Y. (1984). Electrical Resistivity of Selected Elements. J. Phys. Chem. Ref. Data.

[110] Scofield J.H., Duda A., Albin D., Ballard B.L., Predecki P.K. (1995). Sputtered Molybdenum Bilayer Back Contact for Copper Indium Diselenide-Based Polycrystalline Thin-Film Solar Cells. Thin Solid Films.

[111] Chen S.-F., Wang S.-J., Lee W.-D., Chen M.-H., Wei C.-N., Bor H.-Y.Y. (2015). Preparation and Characterization of Molybdenum Thin Films by Direct-Current Magnetron Sputtering. Atlas J. Mater. Sci..

[112] Klenk R., Lux-Steiner M.-C., Poortmans J., Arkhipov V. (2006). Chalcopyrite Based Solar Cells. Thin Film Solar Cells: Fabrication, Characterization and Applications.

[113] Jackson P., Hariskos D., Lotter E., Paetel S., Wuerz R., Menner R., Wischmann W., Powalla M. (2011). New world record efficiency for Cu(In,Ga)Se_2_ thin-film solar cells beyond 20%. Prog. Photovolt. Res. Appl..

[114] Jackson P., Wuerz R., Hariskos D., Lotter E., Witte W., Powalla M. (2016). Effects of heavy alkali elements in Cu(In,Ga)Se_2_ solar cells with efficiencies up to 22.6%. Phys. Status Solidi RRL.

[115] Kamada R., Yagioka T., Adachi S., Handa A., Tai K.F., Kato T., Sugimoto H. New World Record Cu(In,Ga)(Se,S)_2_ Thin Film Solar Cell Efficiency Beyond 22%. Proceedings of the IEEE 43rd Photovoltaic Specialists Conference (PVSC).

[116] Kato T., Wu J.-L., Hirai Y., Sugimoto Y.H., Bermudez V. (2019). Record Efficiency for Thin-Film Polycrystalline Solar Cells Up to 22.9% Achieved by Cs Treated Cu(In,Ga)(Se,S)_2_. IEEE J. Photovolt..

[117] Nakamura M., Yamaguchi K., Kimoto Y., Yasaki Y., Kato T., Sugimoto H. (2019). Cd-Free Cu(In,Ga)(Se,S)_2_ Thin-Film Solar Cell With Record Efficiency of 23.35%. IEEE J. Photovolt..

[118] Mei H., Koch A., Wan C., Rensberg J., Zhang Z., Salman J., Hafermann M., Schaal M., Xiao Y., Wambold R. (2022). Tuning carrier density and phase transitions in oxide semiconductors using focused ion beams. Nanophotonics.

